# Sulfonoquinovosyl diacylglyceride selectively targets acute lymphoblastic leukemia cells and exerts potent anti-leukemic effects *in vivo*

**DOI:** 10.1038/srep12082

**Published:** 2015-07-20

**Authors:** Chetan Kumar Jain, Bhola Shankar Pradhan, Sukdeb Banerjee, Nirup Bikash Mondal, Subeer S. Majumder, Madhumita Bhattacharyya, Saikat Chakrabarti, Susanta Roychoudhury, Hemanta Kumar Majumder

**Affiliations:** 1 Infectious Diseases and Immunology Division, CSIR-Indian Institute of Chemical Biology, 4, Raja S.C. Mullick Road, Jadavpur, Kolkata-700032, India; 2 Cancer Biology and Inflammatory Disorder Division, CSIR-Indian Institute of Chemical Biology, 4, Raja S.C. Mullick Road, Jadavpur, Kolkata-700032, India; 3 Division of Embryo Biotechnology, National Institute of Immunology, New Delhi, 110067, India; 4 Chemistry Division, CSIR-Indian Institute of Chemical Biology, 4, Raja S.C. Mullick Road, Jadavpur, Kolkata-700032, India; 5 Structural Biology and Bioinformatics Division. CSIR-Indian Institute of Chemical Biology, 4, Raja S.C. Mullick Road, Jadavpur, Kolkata-700032, India

## Abstract

DNA topoisomerase II inhibitors *e.g.* doxorubicin and etoposide are currently used in the chemotherapy for acute lymphoblastic leukemia (ALL). These inhibitors have serious side effects during the chemotherapy *e.g.* cardiotoxicity and secondary malignancies. In this study we show that sulfonoquinovosyl diacylglyceride (SQDG) isolated from *Azadirachta indica* exerts potent anti-ALL activity both *in vitro* and *in vivo* in nude mice and it synergizes with doxorubicin and etoposide. SQDG selectively targets ALL MOLT-4 cells by inhibiting catalytic activity of topoisomerase I enzyme and inducing p53 dependent apoptotic pathway. SQDG treatment induces recruitment of ATR at chromatin and arrests the cells in S-phase. Down-regulation of topoisomerase I or p53 renders the cells less sensitive for SQDG, while ectopic expression of wild type p53 protein in p53 deficient K562 cells results in chemosensitization of the cells for SQDG. We also show that constant ratio combinations of SQDG and etoposide or SDQG and doxorubicin exert synergistic effects on MOLT-4 cell killing. This study suggests that doses of etoposide/doxorubicin can be substantially reduced by combining SQDG with these agents during ALL chemotherapy and side effects caused can be minimized. Thus dual targeting of topoisomerase I and II enzymes is a promising strategy for improving ALL chemotherapy.

Acute lymphoblastic leukemia (ALL) is the most common form of leukemia in children between the ages of 2 and 5 years. ALL also affects adults especially people with the age of 65 or older. Survival rate after treatment is 80% in children but in adults it is only 40%^1^,^2^. Common treatment for ALL is combination chemotherapy consisting three different phases *viz*., remission induction, consolidation/intensification and maintenance therapy. Unfortunately, chemotherapeutic drugs for the treatment have serious side effects. For instance, doxorubicin and etoposide (DNA topoisomerase II inhibitors) are currently used in combination with other anti-ALL agents during remission induction and consolidation phases of the chemotherapy, respectively. Doxorubicin treatment shows severe cardiotoxicity while etoposide treatment often induces therapy related secondary malignancies^3^,^4^. Therefore, development of effective, selective and safe anti-ALL chemotherapeutic agents is justified to improve the ALL chemotherapy.

DNA topoisomerases are well established molecular targets of several anticancer and antibacterial drugs and currently many topoisomerase inhibitors are used in the chemotherapy of different cancers^5^,^6^. Topoisomerases control DNA topology by transiently cleaving and religating DNA strands, during various DNA metabolic processes. In the nucleus of mammalian cells two principal types of topoisomerases are present: topoisomerase I (topo I) and topoisomerase II (topo II)^7^,^8^. Enzymatic reaction cycle of topo I proceeds through four different steps- (a) DNA binding: the enzyme binds on its preferential binding site, (b) DNA nicking: nucleophilic attack on phosphodiester backbone by the active site tyrosine-723 residue and formation of transient 3′ phosphotyrosyl bond, (c) controlled strand rotation and (d) religation of DNA strand. Under normal conditions the nicking of DNA strand is transient and nick is rapidly sealed after strand rotation. Trapping of the enzyme and DNA in nicked condition by topo inhibitors *i.e*. stabilization of covalent enzyme-DNA complexes, generates DNA lesions, initiates cell cycle arrest and ultimately induces apoptosis. Several inhibitors are known for topoisomerase enzymes and are classified into two categories: (a) the catalytic topoisomerase inhibitors which inhibit catalytic activity of the enzymes prior to DNA binding and (b) the topoisomerase poisons which inhibit either controlled strand rotation or religation step of enzymatic reaction cycle and thus stabilizes covalent enzyme-DNA complexes^9^. Camptothecin (CPT) is an alkaloid isolated from the bark of the Chinese tree, *Camptotheca acuminate*^10^. CPT stabilizes topo I-DNA covalent complexes by inhibiting the religation step^11^.

To attain high proliferation, cancer cells necessitate higher activity of topoisomerases and indeed these enzymes have been shown to overexpress in several tumors and cancer cell lines^12^,^13^, *e.g.* topo I is overexpressed in ALL cells and also in MOLT-4 cells^12^,^14^. p53 is involved in multicellular processes e.g. cell cycle arrest, senescence, apoptosis and DNA repair^15^. During DNA damage p53 initiates two specific responses *viz*. cell cycle checkpoint activation and cell death via apoptosis. The prerequisite for each of these functions appears to depend on: the cell type, the cell environment and other cellular genetic alterations^16^. p53 plays important role in chemosensitivity and chemoresistance of cancer cells^17^. Mutations in the *TP53* gene often lead to drug resistance, while wild type p53 protein plays important role in chemosensitivity of anti-cancer agents^18^.

Sulfonoquinovosyl diacylglyceride (SQDG) is a member of plant sulfolipids. SQDG was first reported in photosynthetic bacteria and higher plants by Benson and coworkers^19^. SQDG used in the present study was isolated by chromatographic separation of methanolic extract of the leaves of *A. indica* and characterized by extensive 2D-NMR and mass spectroscopy (Fig. 1a)^20^. SQDG has been reported for its anti-leukemic, anti-bacterial and anti-viral activities^20^,^21^. In this study we show that SQDG inhibits topo I enzyme of MOLT-cells, generates DNA replication stress, arrests the cells in S-phase and induces p53 dependent apoptotic pathway. Combinations of SQDG with etoposide and doxorubicin exert synergism and SQDG treatment reduces tumor growth in the nude mice xenografted with MOLT-4 cells.

## Results

### SQDG catalytically inhibits topo I enzyme and prevents camptothecin mediated formation of topo I-DNA covalent complexes *in vitro*

**Figure 1:**
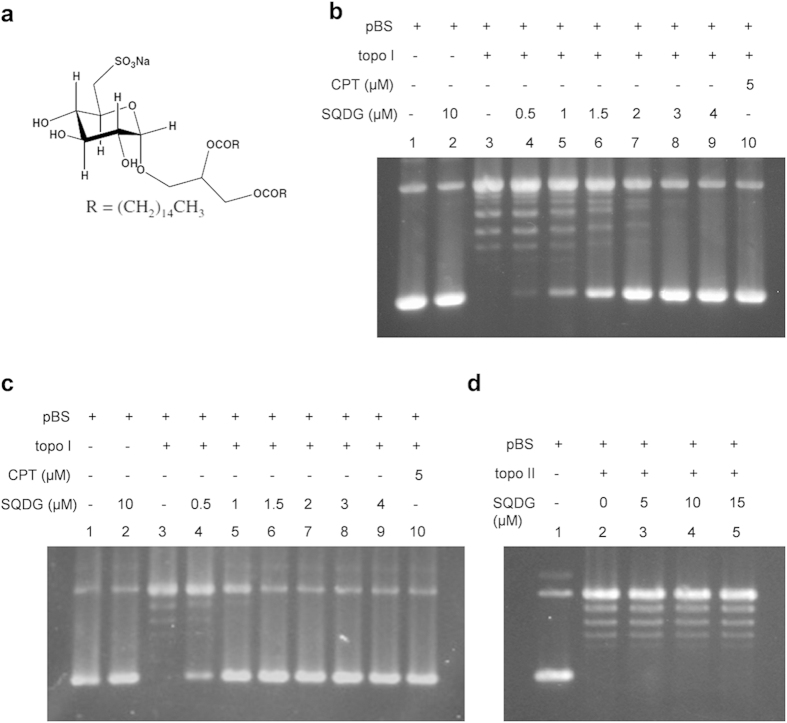
SQDG inhibits relaxation activity of human topoisomerase I enzyme. (**a**) Chemical structure of Sulfonoquinovosyl Diacylglyceride (SQDG). (**b**) DNA relaxation assay of topo I enzyme. Supercoiled pBS DNA was treated with topo I enzyme in the absence or presence of indicated concentrations of SQDG. CPT was used as control inhibitor. Lane 1, 100 fmol pBS DNA; lane 2, 100 fmol pBS DNA with 10 μM SQDG; lane 3, 100 fmol pBS DNA with 50 fmol of topo I enzyme; lanes 4 to 9, same as lane 3 but in the presence of indicated concentrations of SQDG; lane 10, same as lane 3 but in the presence of 5 μM CPT. Reactions were incubated at 37 °C for 30 minutes. (**c**) Preincubation DNA relaxation assay. Topo I was preincubated with indicated concentrations of SQDG or CPT for 5 minutes and then supercoiled pBS DNA was added. All the other conditions were same as DNA relaxation assay. (**d**) DNA relaxation assay of topo IIα enzyme. Lane 1, 100 fmol pBS DNA; lane 2, 100 fmol pBS DNA with 50 fmol of topo IIα enzyme; lanes 3 to 5, same as lane 2 but in the presence of indicated concentrations of SQDG. Complete scans of the different gels are presented in the Supplementary Figure S7.

In order to check the inhibitory effect of SQDG on topo I enzymatic activity, *in vitro* DNA relaxation assay was performed using topo I enzyme and supercoiled pBS DNA in the presence of different concentrations of SQDG. At 3 μM SQDG concentration, complete inhibition of the topo I relaxation activity was observed (Fig. 1b). Preincubation of the enzyme with SQDG for 5 minutes, before adding supercoiled pBS DNA, markedly enhanced the inhibition and relaxation activity was completely inhibited at 1.5 μM SQDG (Fig. 1c). Preincubation dilution assay was also performed to make sure that SQDG bound form of the enzyme is inactive. After 5 minutes preincubation of the enzyme with SQDG, reaction mixture was diluted to 10 folds with the reaction buffer. After the dilution supercoiled DNA was added and relaxation assay was performed (Supplementary Fig. S1). Dilution of the reaction mixtures did not affect topo I inhibition caused by SQDG suggesting that SQDG bound form of the enzyme remains inactive. On the other hand SQDG did not inhibit DNA topoisomerase II alpha enzyme at 5, 10 and 15 μM concentrations (Fig. 1d).

**Figure 2:**
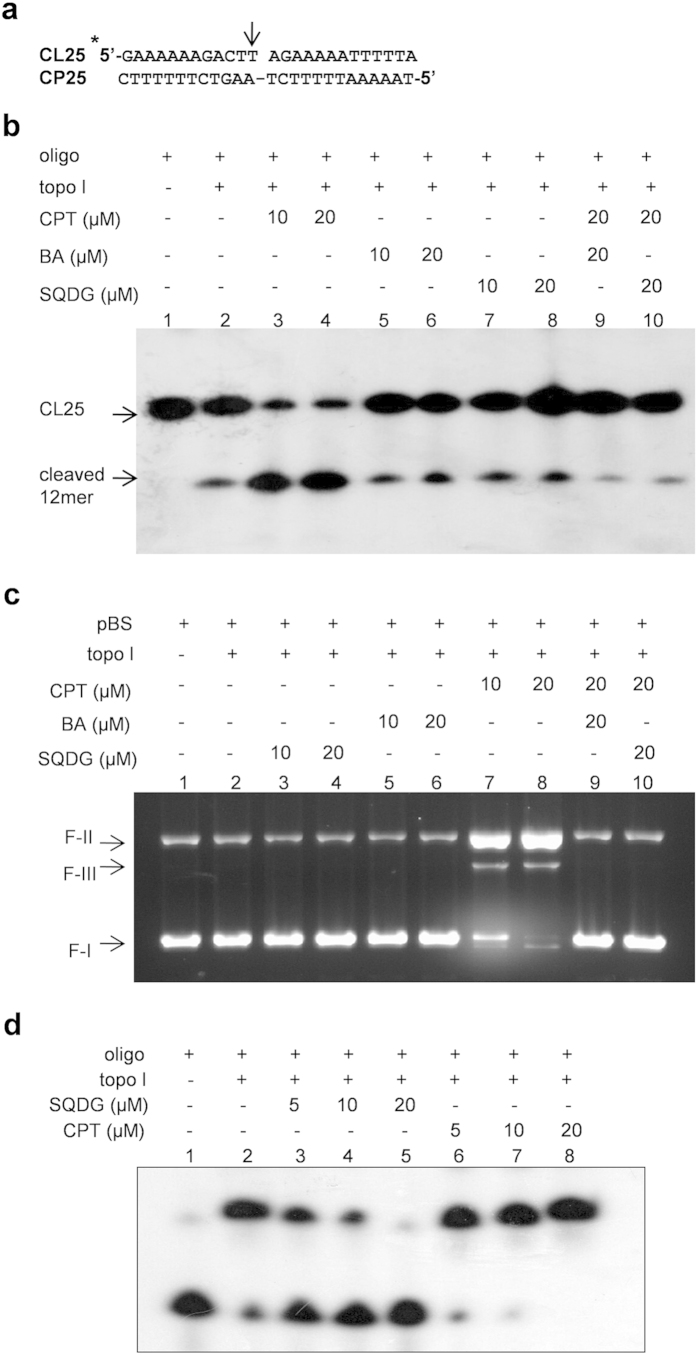
SQDG abrogates CPT mediated topo I-DNA complex formation *in vitro* and inhibits DNA binding of topo I (**a**) Structure of labeled 25-mer duplex oligo used for oligonucleotide cleavage assay. CL25 is the strand to be cleaved while CP25 is the complementary strand. CL25 is labeled with γ-^32^Phosphate at 5′-end, shown with asterisk (*). Arrow (↓) represents the topo I cleavage site. (**b**) Oligonucleotide cleavage assay. Lane 1, 1 pmol of the labeled 25-mer duplex oligo; lane 2, 1 pmol of the labeled 25-mer duplex oligo with 20 ng topo I enzyme; lanes 3 to 10, same as lane 2 but with indicated compounds. Reactions were denatured and separated by 7 M-urea-PAGE (20%) and visualized by autoradiography. (**c**) DNA cleavage assay. Lane 1, 100 fmol of pBS DNA; lane 2, 100 fmol of pBS DNA with 500 fmol of topo I enzyme; lanes 3 to 10, same as lane 2 but with indicated compounds. Reactions were incubated at 37 °C for 30 minutes and stopped with 0.5% SDS. (**d**) Electrophoretic mobility shift assay. Lane 1, 1 pmol of the labeled 25-mer duplex oligo; lane 2, 1 pmol of the labeled 25-mer duplex oligo with 20 ng topo I enzyme; lanes 3 to 8 are same as lane 2 but in the presence of indicated concentrations of SQDG and CPT. Complete scans of the autoradiogram and gel are presented in the Supplementary Figure S8 and S9.

To find whether SQDG is catalytic inhibitor or topo I poison, *in vitro* oligonucleotide cleavage assay was performed using synthetic oligonucleotide with topo I binding site (Fig. 2a). Camptothecin (CPT) is a topo I poison which inhibits religation step of the enzymatic reaction cycle and stabilizes covalent topo I-DNA complexes^6^,^11^. Oligonucleotide cleavage assay in the presence of 10 μM and 20 μM CPT substantially increased the amount of cleaved 12 mer oligo compared to control, as CPT stabilizes topo I-oligo covalent complexes (Fig. 2b). Betulinic acid (BA) is a catalytic inhibitor of topo I^22^,^23^ and was used as another control. In presence of 10 μM and 20 μM SQDG, we did not observe any increase in the amount of cleaved 12 mer oligo compared to the control reaction, indicating that SQDG is not a topo I poison like CPT but it is a catalytic topo I inhibitor like BA. When 20 μM BA or 20 μM SQDG was added in the reactions and incubated for 5 minutes prior to addition of 20 μM CPT, the amount of cleaved 12 mer oligo substantially reduced in comparison to CPT alone reactions, indicating that SQDG/BA abrogates CPT mediated topo I-oligo covalent complex formation (Fig. 2b). Besides, DNA cleavage assay was also performed to revalidate the results of oligonucleotide cleavage assay. SQDG (10 μM and 20 μM) did not stabilize the topo-DNA complexes while CPT (10 μM and 20 μM) substantially increased formation of the topo-DNA complexes (Fig. 2c). The assay further confirmed that SQDG is a catalytic topo I inhibitor. In order to study effect of SQDG on the DNA binding of topo I enzyme electrophoretic mobility shift assay was performed using synthetic oligonucleotide containing topo I binding site (Fig. 2a,d). Results of EMSA indicated that in the presence of 5 and 10 μM SQDG, binding of topo I to oligonucleotide was decreased and in the presence of 20 μM SQDG, binding of topo I to oligonucleotide was almost completely abolished. In the presence of 5, 10 and 20 μM CPT binding of topo I to oligonucleotide was increased which may be due to the stabilization of covalent topo I-oligonucleotide complexes by CPT. Thus, EMSA results clearly suggest that SQDG inhibits DNA binding activity of topo I. In order to understand comparative affinities of CPT and SQDG for the enzyme, competition cleavage assays were performed (Supplementary Fig. S2). Upon preincubation of the enzyme with 5, 10 and 20 μM SQDG for 5 minutes, CPT mediated cleavage was decreased in 5 and 10 μM SQDG preincubated conditions and the cleavage was completely abolished in 20 μM SQDG preincubated condition (Supplementary Fig. S2a). On the other hand upon preincubation of the enzyme with 5, 10 and 20 μM CPT for 5 minutes, addition of 20 μM SQDG did not affect the CPT mediated cleavage complex formation (Supplementary Fig. S2b). Besides, upon simultaneous addition of SQDG and CPT, CPT mediated cleavage was not increased in 10 and 20 μM CPT conditions and was same as 5 μM CPT condition (Supplementary Fig. S2c). These results suggest that both SQDG and CPT have similar affinities for the enzyme. Together the results of cleavage assays and electrophoretic mobility shift assay (Fig. 2 and Supplementary Fig. S2) suggest that SQDG inhibits topo I upstream in the enzymatic reaction at the DNA binding step whereas CPT inhibits topo I downstream in the enzymatic reaction at relegation step. Thus, upon preincubation of the enzyme is with SQDG, CPT mediated cleavage is not observed and opposite to this, upon preincubation of the enzyme with CPT, SQDG can not affect CPT mediated cleavage complex formation. However, upon simultaneous addition of SQDG and CPT, CPT mediated cleavage is observed but the cleavage does not increase with increasing concentrations of CPT, suggesting that in the presence of SQDG the enzyme becomes unable to bind to DNA and therefore CPT can not stabilize covalent enzyme-DNA complexes anymore.

### SQDG selectively kills acute lymphoblastic/lymphocytic leukemia cell lines and the cell killing is topo I dependent

**Figure 3:**
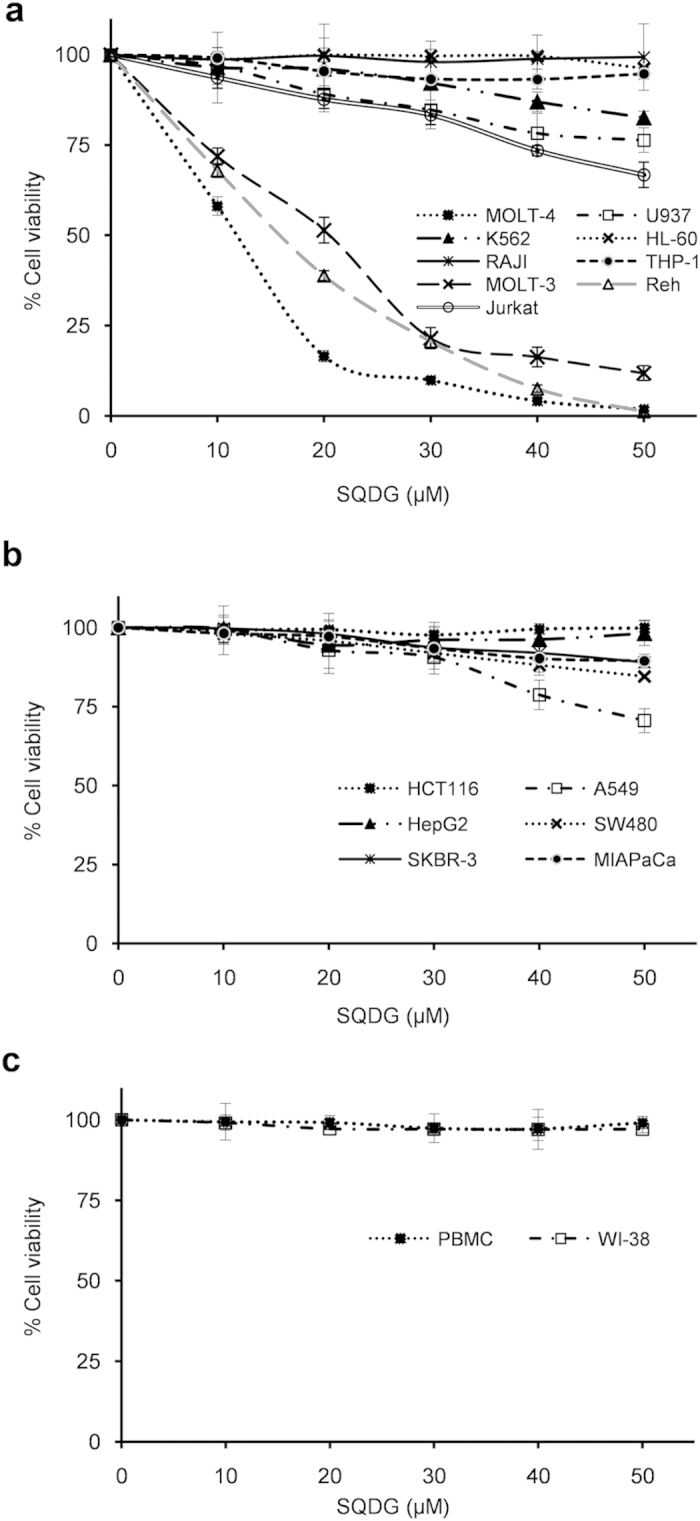
Effect of SQDG treatment on viability of different cell lines. (**a**) Nine different leukemia cell lines treated with indicated concentrations of SQDG for 72 hours. Three independent experiments were performed and data are represented as mean % cell viability ± SD. (**b**) Six different solid tumor cell lines treated with indicated concentrations of SQDG for 72 hours. Three independent experiments were performed and data are represented as mean % cell viability ± SD. (**c**) PBMC and WI-38 cells treated with indicated concentrations of SQDG for 72 hours. Three independent experiments were performed and data are represented as mean % cell viability ± SD.

Cell viability experiments indicated that SQDG selectively induces killing of ALL cell lines: MOLT-4, MOLT-3 and Reh. IC_50_ values of SQDG for MOLT-4, MOLT-3 and Reh cell lines were found to be 15.32 ± 0.58 μM, 22.52 ± 0.64 μM and 19.63 ± 0.23 μM, respectively. SQDG did not affect the growth of other leukemic cell lines e.g. RAJI, THP-1 and HL-60. However, K562 and U937 cells were partially affected at 50 μM concentration (Fig. 3a and Supplementary Table S1). Jurkat cell line was also found to be resistant for SQDG treatment with a very high IC_50_ value of 75.67 ± 6.4 μM. One difference among MOLT-4, MOLT-3, Reh and the other leukemic cells used in this study is p53 status. Studies have shown that MOLT-4, MOLT-3 and Reh cell lines express wild type p53^24^,^25^,^26^,^27^; Jurkat, RAJI, THP-1 and U937 cells express mutant p53^28^,^29^,^30^ while K562 and HL-60 are devoid of p53 protein^31^,^32^,^33^. Therefore, the effects of SQDG treatment were also checked on three p53 wild type solid tumor cell lines and three p53 mutant solid tumor cell lines. Surprisingly, SQDG did not affect viability of the cell lines and only A549 cells were partially affected at 50 μM concentration of SQDG. SQDG also did not affect viability of WI-38 cells (normal lung fibroblast cell line) and peripheral blood mononuclear cells (PBMC) (Fig. 3b,c and Supplementary Table S1).

**Figure 4:**
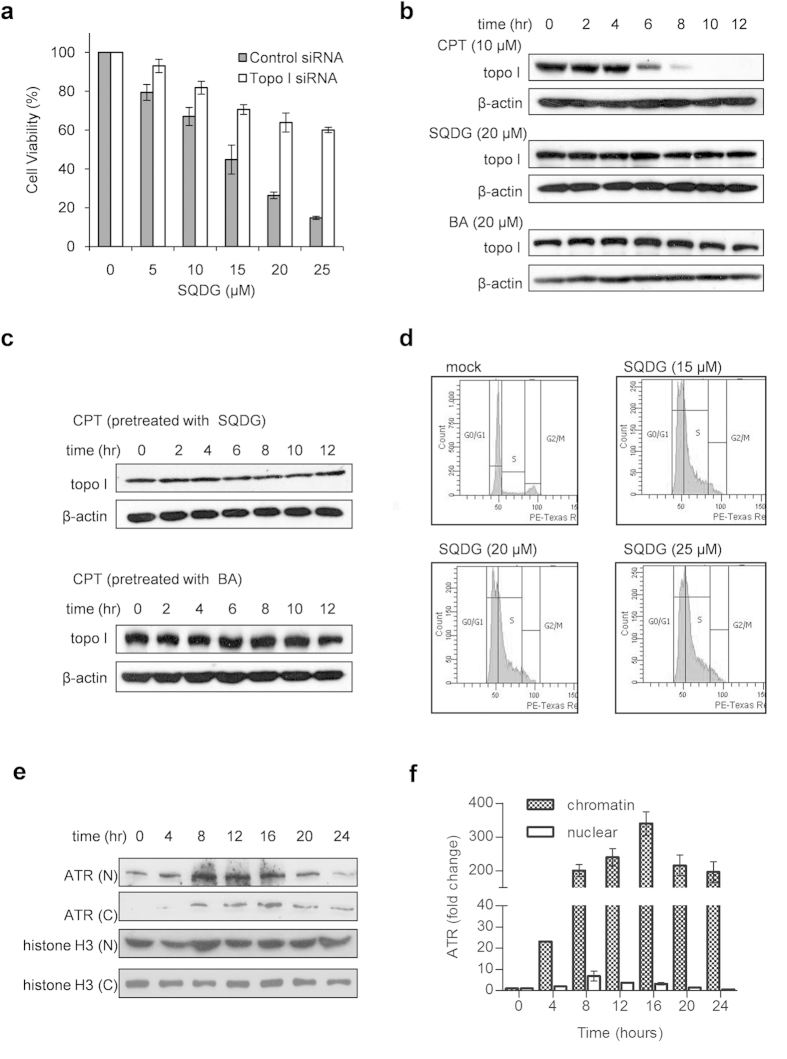
SQDG mediated MOLT-4 cell killing is topo I dependent. SQDG treatment precludes camptothecin mediated formation of topo I-DNA complexes and generates DNA replication stress in MOLT-4 cells. (**a**) Knockdown of topo I in MOLT-4 cells. MOLT-4 cells were transfected with 100 nM topo I siRNA or 100 nM control siRNA (ctrl siRNA). After 48 hours of transfection cells were treated with indicated concentrations of SQDG for 72 hours and MTT assay was performed. Three independent experiments were performed and data are represented as mean % cell viability ± SD. Solid bars indicate cells transfected with control siRNA and hollow bars indicate cells transfected with topo I siRNA. (**b**) Topo I immunoband depletion assay in MOLT-4 cells. The cells were treated with 10 μM CPT or 20 μM SQDG or 20 μM BA and harvested at indicated time points. Western blotting was performed using anti-topo I or anti-β-actin antibodies. Complete scans of the different blots are presented in the Supplementary Figure S11. (**c**) Pretreatment immunoband depletion assay. MOLT-4 cells were first treated with either 20 μM SQDG or 20 μM BA for 2 hours and then treated with 10 μM CPT for indicated time points. Western blotting was performed using anti-topo I or anti-β-actin antibodies. Complete scans of the different blots are presented in the Supplementary Figure S12. (**d**) Cell cycle analysis of SQDG treated MOLT-4 cells. Cells were treated with different concentrations of SQDG (15 μM, 20 μM and 25 μM) for 24 hours. Cells were fixed in 70% ethanol and flow cytometry was performed. (**e**) ATR recruitment at chromatin in SQDG treated MOLT-4 cells. MOLT-4 cells were treated with 15 μM SQDG for indicated time points and nuclear and chromatin fractionations were performed. Levels of ATR in chromatin and nuclear fractions were detected by western blot analysis. Histone-H3 was used as loading control. ‘N’ stands for nuclear fraction and ‘C’ stands for chromatin fraction. Complete scans of the different blots are presented in the Supplementary Figure S13. (**f**) Histogram showing fold changes of ATR in chromatin and nuclear fractions after 15 μM SQDG treatment for indicated time points. Protein levels were quantitated by densitometry analysis of gel bands. ATR levels were normalized with respective histone H3 levels and fold change was calculated. Error bars show standard deviation of mean for the two independent experiments.

To assess the role of topo I inhibition in SQDG mediated killing of MOLT-4 cells, siRNA silencing of *TOP1* gene was performed by using a pool of three different siRNAs. Knockdown of *TOP1* gene in MOLT-4 cells rendered the cells less sensitive for SQDG treatment (Fig. 4a and Supplementary Fig. S3a). IC_50_ value of SQDG for control siRNA transfected cells was found to be 14.04 ± 0.71 μM while IC_50_ value of SQDG for topo I siRNA transfected cells was found to be 29.09 ± 2.08 μM (Supplementary Table S2). Fold resistance was calculated by the ratio of IC_50_ value of siRNA transfected cells to IC_50_ value of control siRNA transfected cells. Fold increase in resistance for topo I siRNA transfected cells was found to be 2.07 folds. Knockdown of *TOP1* gene was corroborated by one additional siRNAs pool to mitigate off-target effects (Supplementary Fig S4a. and Supplementary Table S2). siRNA silencing of *TOP1* gene was also performed in Reh cell line. Similar to MOLT-4 cells in Reh cells knockdown of *TOP1* gene rendered the cells less sensitive for SQDG treatment (Supplementary Fig S4b and S4c). IC_50_ value of SQDG for control siRNA transfected cells was found to be 14.48 ± 0.28 μM while IC_50_ value of SQDG for topo I siRNA transfected cells was found to be 27.51 ± 0.41 μM (Supplementary Table S2). Fold increase in resistance for topo I siRNA transfected cells was found to be 1.89 folds. These results indicate that SQDG mediated MOLT-4 and Reh cell killings are topo I dependent.

### SQDG treatment precludes camptothecin mediated formation of topo I-DNA complexes in MOLT-4 cells

Topo I poisons, which trap the topo I-DNA covalent complexes, upon addition to the cells deplete the immunoband of topo I enzyme. When MOLT-4 cells were treated with 10 μM CPT or 20 μM SQDG for different time points and immunoblotting for topo I was performed, CPT started depletion of topo I immunoband at 6 hours time point and completely depleted the immunoband at 10 hours of treatment (Fig. 4b, panel for CPT). SQDG as speculated from *in vitro* experiments did not deplete the topo I immunoband even at 12 hours of the treatment (Fig. 4b, panel for SQDG). Treatment with 20 μM BA also did not deplete immunoband of topo I (Fig. 4b, panel for BA). Moreover, pretreatment of MOLT-4 cells with 20 μM SQDG for 2 hours before treating with 10 μM CPT completely abrogated the immunoband depletion mediated by CPT, indicating that SQDG inhibits catalytic activity of the enzyme (Fig. 4c, upper panel). Same results were observed when the cells were pretreated with 20 μM BA for 2 hours before treating with 10 μM CPT (Fig. 4c, lower panel). Thus SQDG treatment of MOLT-4 cells inhibits the catalytic activity of topo I and CPT cannot stabilize the topo I-DNA covalent complexes.

### SQDG treatment induces DNA replication stress and activates p53 dependent apoptotic cell death pathway in MOLT-4 cells

Cell cycle arrest analysis by flow cytometry showed that treatment of MOLT-4 cells with 15, 20 and 25 μM SQDG for 24 hours increased the S-phase population in the dose dependent manner (Fig. 4d and Supplementary Table S3). Since SQDG treatment arrested cells in S-phase and studies have shown that inhibition of topo I impairs DNA replication as topo I has profound role during cellular DNA replication^34^,^35^, therefore we suspected that SQDG treatment may induce DNA replication stress as a result of topoisomerase I inhibition. In order to test the induction of replication stress we checked recruitment of ATM-and-Rad3-related kinase (ATR) at chromatin after SQDG treatment. ATR is known to be recruited at chromatin during the replication stress as stalled replication forks recruit ATR^36^,^37^,^38^. In response to SQDG treatment we observed ATR enrichment at chromatin, reflecting the generation of DNA replication stress (Fig. 4e,f and Supplementary Table S4). After 4 hours of the treatment, 23 folds increased in ATR recruitment at chromatin was observed which increased to 315 folds after 16 hours of SQDG treatment, indicating the generation of replication stress in SQDG treated cells. Recruitment of ATR at chromatin was gradually decreased in 20 and 24 hours and was 194 and 174 folds, respectively.

**Figure 5:**
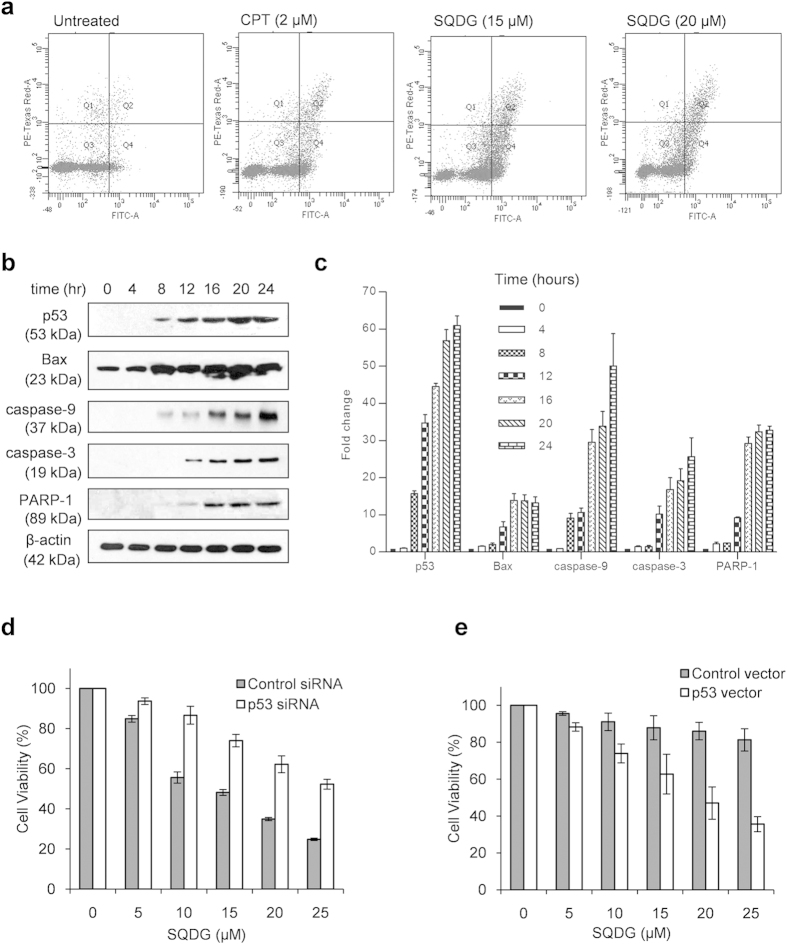
SQDG treatment induces p53 dependent apoptotic pathway in MOLT-4 cells and ectopic expression of p53 in K562 cells sensitizes the cells for SQDG. (**a**) FITC-annexin V-propidium iodide staining of MOLT-4 cells untreated or treated with CPT or SQDG. Cells were treated with either CPT (2 μM) or SQDG (15 μM or 20 μM) for 48 hours. (**b**) Analysis of p53 dependent apoptotic pathway. MOLT-4 cells were treated with 15 μM SQDG for indicated time points and immunoblotting was performed using specific antibodies for indicated proteins involved in p53 dependent pathway. Complete scans of the different blots are presented in the Supplementary Figure S14. (**c**) Fold changes of levels of proteins involved in p53 dependent pathway upon treatment with 15 μM SQDG for indicated time points. Protein levels were quantitated by densitometry analysis of gel bands. Protein levels were normalized with respective β-actin levels and fold change was calculated. Error bars show standard deviation of mean for two independent experiments. (**d**) Knockdown of p53 in MOLT-4 cells. MOLT-4 cells were transfected with 100 nM p53 siRNA or 100 nM control siRNA. After 72 hours of transfection cells were treated with indicated concentrations of SQDG for 72 hours and MTT assay was performed. Three independent experiments were performed and data are represented as mean % cell viability ± SD. Solid bars indicate cells transfected with control siRNA and hollow bars indicate cells transfected with p53 siRNA. (**e**) Ectopic expression of p53 in p53 deficient K562 myeloid leukemia cells. K562 cells were transfected with 400 ng control vector or 400 ng p53 expressing vector pCMV-NEO-BAM. After 36 hours of transfection cells were treated with indicated concentrations of SQDG for 72 hours and MTT assay was performed. Three independent experiments were performed and data are represented as mean % cell viability ± SD. Solid bars indicate cells transfected with control vector and hollow bars indicate cells transfected with p53 expressing vector.

To study whether SQDG induces apoptosis in MOLT-4 cells, we performed annexin-V and propidium iodide staining of SQDG treated and untreated cells followed by flow cytometric analysis. CPT (2 μM) was used as a positive control for apoptosis induction. Treatment of MOLT-4 cells with 15 μM or 20 μM SQDG for 48 hours markedly induced apoptosis in MOLT-4 cells (Fig. 5a). Since MOLT-4 cells express wild type p53, therefore we thought that p53 might play role in induction of apoptosis in these cells. In order to study p53 dependent apoptotic pathway, MOLT-4 cells were treated with 15 μM SQDG for different time points and induction of the proteins involved in p53 dependent apoptotic pathway was checked (Fig. 5b,c). A 34 fold increase in p53 activation was observed after 8 hours of SQDG treatment, which reached to 60 folds at 24 hours post-treatment. Bcl-associated X protein (Bax) activation is associated with induction of p53 dependent apoptosis. Bax is a transcriptional target of p53 and it induces cytochrome c release from mitochondrial membrane^39^,^40^. Bax was activated by 2 folds after 8 hours of SQDG treatment and increased to 13 folds at 24 hours post-treatment. Cytochrome c together with apoptotic protease activating factor-1 and procaspase-9 forms a complex called apoptosome, which cleaves procaspase-9 and converts it in to caspase-9^41^,^42^. Cleavage of procaspase-9 was increased by 9 folds after 8 hours of SQDG treatment and reached to 50 folds at 24 hours post-treatment. Caspase-9 cleaves procaspase-3 into caspase-3, which initiates process of cell death^41^. Caspase-3 is an executive caspase which activates various endonucleases and proteases. During apoptosis induction PARP-1, a 113 kDa protein, is cleaved into 89 and 24 kDa fragments. In our study caspase-3 and PARP-1 cleavages started at 12 hours of SQDG treatment and continued up to 24 hours of the treatment. Together these findings suggest that SQDG treatment induces a p53 dependent apoptotic pathway in MOLT-4 cells. To further study p53 dependency of SQDG mediated cell killing, siRNA gene silencing was performed to knockdown *TP53* gene expression in MOLT-4 cells (Fig. 5d and Supplementary Fig. S3b). Cell viability experiments with *TP53* silenced cells indicated that knockdown of p53 renders the cells less sensitive for SQDG treatment. IC_50_ value of SQDG for control siRNA transfected cells was 15.15 ± 0.13 μM while for p53 siRNA transfected cells it became 26.85 ± 2.08 μM (Supplementary Table S5). Fold increase in resistance for p53 siRNA transfected cells was found to be 1.77 folds. Knockdown of *TP53* gene was corroborated by one additional siRNAs pool to mitigate off-target effects (Supplementary Fig S4d. and Supplementary Table S5). siRNA silencing of *TP53* gene was also performed in Reh cell line. Similar to MOLT-4 cells in Reh cells knockdown of *TP53* gene rendered the cells less sensitive for SQDG treatment (Supplementary Fig S4e and S4f). IC_50_ value of SQDG for control siRNA transfected cells was found to be 14.31 ± 1.04 μM while IC_50_ value of SQDG for p53 siRNA transfected cells was found to be 22.35 ± 2.42 μM (Supplementary Table S5). Fold increase in resistance for p53 siRNA transfected cells was found to be 1.56 folds. Knockdown of *TP53* gene was corroborated by one additional siRNAs pool to mitigate off-target effects (Supplementary Fig S4f). On the other hand ectopic expression of p53 protein in p53 deficient K562 myeloid leukemia cells amended these cells relatively more sensitive for SQDG treatment (Fig. 5e and Supplementary Fig. S3c). IC_50_ value of SQDG for K562 cells transfected with empty vector was found to be 56.51 ± 1.14 μM while IC_50_ value of SQDG for K562 cells ectopically expressing wild type p53 was found to be 19.54 ± 2.51 μM (Supplementary Table S6). Fold increase in sensitivity for K562 cells ectopically expressing p53 was found to be 2.89 folds. p53 protein was also ectopically expressed in an another p53 deficient cell line: HL-60. Similar to K562 cells ectopic expression of p53 protein in HL-60 cells amended these cells relatively more sensitive for SQDG treatment (Supplementary Fig S5). IC_50_ value of SQDG for the cells transfected with empty vector was found to be 92.65 ± 6.35 μM while IC_50_ value of SQDG for the cells ectopically expressing wild type p53 was found to be 24.01 ± 2.0 μM (Supplementary Table S6). Fold increase in sensitivity for HL-60 cells ectopically expressing p53 was found to be 3.85 folds. Together these observations suggest that SQDG mediated leukemic cell killing is p53 dependent.

### SQDG synergizes with etoposide and doxorubicin

Dose-effect curves for etoposide-SQDG and doxorubicin-SQDG drug combinations indicated that the combinations exert synergism as all the data points for the two combinations fell very near to Fa value of 1.0, (Supplementary Fig. S6a and S6b). As dose-effect curves do not provide any quantitative measurement of synergy therefore using CompuSyn software median-effect analysis was performed to quantitatively evaluate etoposide-SQDG and doxorubicin-SQDG drug combinations^43^,^44^,^45^. In median-effect analysis a combination index value (CI) is calculated for each drug combination. CI = 1.0, is additive effect; CI > 1.0, is antagonism and CI < 1.0, is synergism. CI values for etoposide-SQDG and doxorubicin-SQDG combinations ranged from 0.4 to 0.7 and from 0.1 to 0.6, respectively (Tables 1 and 2). These CI values are indicative of synergism and demonstrate that etoposide-SQDG and doxorubicin-SQDG combinations exert synergistic effects on MOLT-4 cell killing. In the Table 2, for combination of doxorubicin (1.4 μM) and SQDG (35 μM), a sudden drop in the CI value (CI value = 0.136 ± 0.036) was observed. It has been shown that topo IIα isoform shows sensitivity for both doxorubicin and etoposide whereas topo IIβ isoform is more sensitive for doxorubicin than etoposide^46^. Thus, sudden drop in the CI value for the combination of doxorubicin (1.4 μM) and SQDG (35 μM) is due to inhibition of both the isoforms of topo II enzyme by doxorubicin and inhibition of topo I by SQDG. In the isobolograms, generated using CompuSyn software Fa values for etoposide-SQDG and doxorubicin-SQDG combinations fell below their corresponding straight lines, which further indicated synergism of the combinations (Supplementary Fig. S6c and S6d).

### SQDG inhibits tumor growth in nude mice

**Figure 6:**
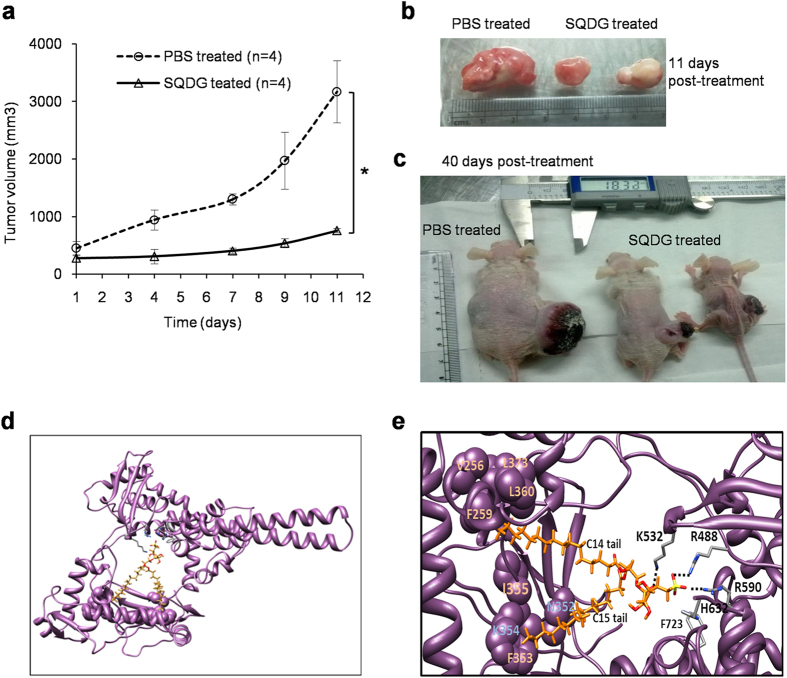
Effects of SQDG treatment on growth of MOLT-4 subcutaneous xenografts in nude mice and molecular docking of SQDG with topo I enzyme. (**a**) Plot for tumor volume versus treatment time. SQDG (2 mg/kg/body weight) was injected intraperitoneally in the mice bearing MOLT-4 xenografts (*n = 4*). In control mice equal volumes of PBS were injected (*n = 4*). Treatments were given at day 1 and day 7 and tumor volumes were measured at day 1, day 4, day 7, day 9 and day 11. Each data point represents the average tumor volume. Data were analyzed by Student’s T-test and were found to be statistically significant (*P* < 0.05, SQDG vs. PBS treated). (**b**) Image of tumors from SQDG treated and vehicle treated mice at day 11 post start of the treatment. (**c**) Image of mice bearing tumors in SQDG treated and vehicle treated groups at day 40 post stop of the treatment. (**d**) SQDG (shown in orange) docks in the DNA binding region of topo I protein (PDB ID: 1A36) (shown in purple). (**e**) Predicted interactions between SQDG and topo I. Oxygen and sulfur atoms of SQDG are colored as red and yellow, respectively. Possible hydrogen bonds between the oxygen atoms and amino acid residues are shown as dotted lines. The amino acid residues in vicinity with hydrophobic carbon tails of SQDG are shown as spheres (purple).

*In vivo* study in nude mice demonstrated that SQDG exerts potent antitumor activity and significantly inhibits growth of tumors. In control group, MOLT-4 xenografted mice (n = 4) were injected with PBS only. In SQDG treated group, MOLT-4 xenografted mice (n = 4) were injected with 2 mg/kg body weight SQDG once per week. In the SQDG treated mice, tumor volumes were significantly reduced compared to PBS treated mice (Fig. 6a–c). Mean tumor doubling time (TD) for the PBS treated mice was 4 ± 0.5 days, which upon SQDG treatment significantly increased to 11.5 ± 2 days (values are mean ± SEM; *P* < 0.05, SQDG vs. PBS treated). Specific tumor growth delay (SGD), which represents the number of tumor doubling times delayed by the treatment^47^, was found to be 1.87 in response to SQDG treatment, indicating that the treatment delayed TD by 1.87 times compared to PBS treatment.

In order to assess tumor regression pharmaco-dynamic (PD) markers in the tumor tissues from vehicle and SQDG treated mice were analyzed (Supplementary Table S9). For assessment of apoptosis in the tumor cells, cleavage of PARP-1 was analysed by immunohistochemical method using specific antibody to detect cleaved PARP-1. Cleavage of PARP-1 was found to be at very low levels in the tumor tissues from vehicle treated mice whereas in the tumor tissues from SQDG treated mice PARP-1 cleavage increased to the intermediate levels (Supplementary Table S9), suggesting the induction of apoptosis in the SQDG treated tumor cells. For assessment of S phase arrest in the tumor cells, cyclin A2 expression was analysed by immunohistochemical method. Cyclin A2 expression was found to be at intermediate level in the tumor tissues from vehicle treated mice whereas in the tumor tissues from SQDG treated mice cyclin A2 expression increased to the high levels (Supplementary Table S9), suggesting S phase arrest in the SQDG treated tumor cells. For assessment of DNA damage response in the tumor cells, γ-H2AX levels were analysed by immunohistochemical method. γ-H2AX levels were found to be low in the tumor tissues from vehicle treated mice whereas in the tumor tissues from SQDG treated mice γ-H2AX levels were increased at intermediate levels (Supplementary Table S9), suggesting induction of apoptotic DNA damage in the SQDG treated tumor cells. In addition to PD markers, expression of Ki-67 (cell proliferation marker) was also analysed in the tumor cells. Ki-67 expression was found to be high in the tumor cells from vehicle treated mice whereas in the tumor cells from SQDG treated mice Ki-67 expression was found to be low (Supplementary Table S9), suggesting that tumor cell proliferation is decreased upon SQDG treatment. Together these results suggest that SQDG treatment induces S phase arrest, apoptosis and apoptotic DNA damage and thereby decreases cell proliferation in the tumor tissues. These results also substantiate the *in vitro* observations for S-phase arrest and apoptosis induction in MOLT-4 cells. Together these results provide substantial evidence that SQDG exerts potent anti-leukemic activity *in vivo* and thus SQDG is a promising anti-leukemic agent.

### SQDG docks in the DNA binding region of topo I by forming non-covalent interactions

Molecular docking was performed between human topoisomerase I (PDB ID: 1A36)^48^ and SQDG (Pubchem, CID 50898453)^20^ (Fig. 6d). The best fit docking solution predicted that the head group of SQDG forms three hydrogen bonds with the active site residues (ARG488, ARG590, LYS532) and the ring oxygen of SQDG forms an electrostatic interaction with the side chain of LYS532 (Fig. 6e). Hydrocarbon tails of SQDG span in the DNA binding groove of the enzyme, probably by forming hydrophobic interactions with various hydrophobic residues in this region (F353, I355 in vicinity with the C15 side chain of SQDG and V256, F259, G359, L360, L373 in vicinity with the C14 side chain of SQDG). Together these results predict that SQDG non-covalently interacts with the enzyme and blocks DNA binding of the enzyme. Docking results also corroborate EMSA results.

## Discussion

This study demonstrates that SQDG isolated from the leaves of *Azadirachta indica* exerts selective and potent anti-ALL effects by inhibiting DNA relaxation activity of topo I. Silencing of *TOP1* gene in MOLT-4 and Reh cell lines increased resistance of these cell lines for SQDG treatment, suggesting that SQDG mediated MOLT-4 and Reh cell killing is mediated by topo I inhibition. Studies have shown that topo I is over expressed in ALL and MOLT-4 cells^12^,^14^. In our study SQDG selectively killed ALL cell lines (MOLT-4, MOLT-3 and Reh) without affecting the growth of other leukemic cells and solid tumor cells. This selective cell killing may be reflected due the higher expression of topo I in these cells. One difference among MOLT-4, MOLT-3, Reh and the other leukemic cells used in this study is p53 status. p53 is well known for inducing cell cycle arrest and/or apoptosis in response to various cellular stresses^49^ and p53 is activated upon genotoxic stresses like DNA damage and DNA replication stress^50^,^51^. During DNA replication stress enrichment of ATR at chromatin activates ATR and activated ATR phosphorylates checkpoint kinase-1 which subsequently activates and stabilizes p53 protein^50^. In our study we also found the enrichment of ATR at chromatin and thus we hypothesized that SQDG treatment may stabilize p53 protein and induce p53 dependent apoptotic pathway following topo I inhibition, DNA replication stress and S-phase arrest in MOLT-4 cells. To test the hypothesis two different experiments were performed. First, *TP53* gene of MOLT-4 cells was knocked down using siRNA. Knocking down p53 increased the resistance of MOLT-4 cells for SQDG. In the second experiment MOLT-4 cells were treated with SQDG for different time points and induction of p53 dependent apoptotic pathway was checked. SQDG treatment stabilized p53 and activated the proteins involved in the p53 dependent apoptotic pathway. These experiments confirmed the involvement of p53 dependent pathway in SQDG mediated cell killing. Involvement of p53 in leukemia cell chemosensitivity has been previously described by Trepel *et al.*^52^. K562 myeloid leukemia cells do not express p53 protein^31^,^33^. In our study K562 cells were merely partially affected by SQDG treatment at more than 50 μM concentration but ectopic expression of wild type p53 protein rendered these cells relatively more sensitive to SQDG treatment. This observation further supported the p53 dependency for SQDG mediated leukemic cell killing. Altogether these observations suggest that overexpression of topo I and expression of wild type p53 in MOLT-4 cells participate to the higher sensitivity of these cells for SQDG. One question which still remains is: why and how SQDG did not affect the growth of solid tumor cell lines which express both topo I and wild type p53 e.g. HCT116, HepG2? One possible reason could be involvement of one or more factors associated with leukemic cells which facilitate(s) the SQDG mediated cell killing. Although, A549 cells, with wild type p53, were partially (~25%) killed at 50 μM SQDG.

*In vivo* study in nude mice demonstrated that SQDG treatment delays tumor doubling time and reduces the expression of cell proliferation marker: Ki67, indicating *in vivo* anti-tumorigenic activity of SQDG. Treatment of mice with SQDG also induced S phase arrest, apoptosis and apoptotic DNA damage in the tumor tissues. Combinations of SQDG with etoposide and doxorubicin exerted synergistic effects on killing of MOLT-4 cells. Thus we suspect that using these combinations, doses of etoposide and doxorubicin can be reduced during ALL chemotherapy and side effects caused by the chemotherapeutic agents can be minimized. Altogether, our study suggests that SQDG is a novel drug candidate for use in ALL chemotherapy and combining the anti-ALL drugs with SQDG might improve the chemotherapeutic regimen for ALL. Selective killing of MOLT-4 cells by SQDG and its *in vivo* anti-tumor activity in nude mice makes it attractive and promising anti-ALL agent. This study indicates the prospect of SQDG as a lead molecule for future ALL chemotherapy.

## Methods

### Reagents, cell culture and cell viability assays

SQDG was isolated as described previously^20^. Etoposide, doxorubicin, betulinic acid and camptothecin were purchased from Sigma (St. Louis, MO, US). Cell lines were cultured in the ATCC recommended culture media supplemented with 10% fetal bovine serum and antibiotics (Invitrogen, Carlsbad, CA, US). Cells were seeded in 96 well plates and treated with respective compounds. After treatment cells were incubated with 3-(4,5-dimethylthiazol-2-yl)-2,5-diphenyltetrazolium bromide (MTT) and colorimetry was performed on Thermo MultiskanEX plate reader at 595 nm.

### Ethics statement

Experiments with nude mice were conducted at National Institute of Immunology (NII). Mice were obtained from the Small Animal Facility of the NII. All animals were housed and used as per the national guidelines provided by the Committee for the Purpose of Control and Supervision of the Experiments on Animals. Protocols for the experiments with nude mice were approved by the Institutional Animal Ethics Committee of NII. All the methods were carried out in accordance with the approved guidelines.

### DNA relaxation assay

Human DNA topoisomerase I and topoisomerase II enzymes were purchased from TopoGEN Inc (Port Orange, Florida, USA). For DNA relaxation assay, 100 fmol supercoiled pBluescript SK(+) (pBS) DNA per reaction was used and the assay was carried out as described previously^22^.

### Oligonucleotide cleavage assay

Synthetic oligonucleotides containing topo I binding site^53^ were purchased from IDT Inc. (San Jose, CA, USA). CL25 oligonucleotide single strand was 5′-end labeled using γ-^32^Phosphate ATP by T4-polynucleotide kinase reaction and CP25 oligonucleotide single strand was 5′-end labeled using ATP. The strands were annealed in annealing buffer [10 mM Tris-HCl (pH 7.5), 1 mM EDTA, and 100 mM NaCl] and oligonucleotide cleavage assay was performed as described previously^23^.

### DNA cleavage assay

DNA cleavage assay was performed as described previously^22^.

### Electrophoretic mobility shift assay

Electrophoretic mobility shift assay for analysis of topo I-oligonucleotide binding was performed as described previously^54^. The same oligonucleotide used for oligonucleotide cleavage assay was used for electrophoretic mobility shift assay.

### Immunoband depletion assay

MOLT-4 cells were treated with 10 μM CPT or 20 μM SQDG or 20 μM BA and harvested at different time points. For pretreatment immunoband depletion assay, cells were first treated with either 20 μM SQDG or 20 μM BA for 2 hours and then treated with 10 μM CPT. Equal amounts of protein were electrophoresed on 8% SDS-poly acryl amide gel and western blotting was performed using anti-topo I antibody (Santa Cruz Biotechnology, Inc. Santa Cruz, CA, USA). Details of the antibody are provided in the Supplementary Table S7.

### Small interfering RNA (siRNA) silencing of topo I or p53 and ectopic expression of p53

Control siRNA, topo I siRNA and p53 siRNA were transfected according to the manufacturer’s protocol. For topo I gene silencing, cells were cultured for 48 hours after the siRNA transfection. For p53 gene silencing, cells were cultured for 72 hours after the siRNA transfection. Details of the siRNAs used are provided in the Supplementary Table S8. For ectopic expression of p53 in K562 and HL-60 cell lines, cells were cultured for 48 hours after transfection with p53 expressing vector: pCMV-NEO-BAM (a kind gift from Dr. Bert Vogelstein)^55^. All transfections were performed using Lipofectamine-2000 reagent (Invitrogen). Gene expression was confirmed by western blot analysis of the respective gene products.

### Flow cytometric detection of apoptosis and cell cycle arrest

For apoptosis detection, MOLT-4 cells were treated with CPT (2 μM) and SQDG (15 μM/20 μM) for 48 hours. Cells were stained using FITC-annexin-V apoptosis detection kit (BD Biosciences, San Diego, CA, USA) and flow cytometry was performed. For cell cycle analysis, MOLT-4 cells were treated with SQDG (15 μM, 20 μM and 25 μM) for 24 hours. Cells were fixed in 70% ethanol, incubated at 4 °C for overnight and stained with PI/RNase solution for flow cytometry analysis.

### Nuclear and chromatin fractionations

Nuclear and chromatin fractionations were performed as described previously^56^. For ATR detection, equal amounts of nuclear and chromatin proteins were electrophoresed and immunoblotting was performed for ATR and histone H3 proteins. For quantitative measurement of ATR enrichment at chromatin, densitometry was performed using ImageJ software. ATR levels were normalized with histone H3 levels and fold change was calculated.

### Apoptotic pathway analysis

MOLT-4 cells were treated with 15 μM SQDG and harvested at 0, 4, 8, 12, 16, 20 and 24 hours. Total protein was isolated from the cells using NP-40 lysis buffer (Invitrogen Corp. CA). Immunoblotting was performed for proteins of p53 dependent apoptotic pathway. Details of the antibodies used are provided in the Supplementary Table S7.

### Drug combination effect analysis

MOLT-4 cells were treated with individual agents or constant ratio combinations of etoposide-SQDG and doxorubicin-SQDG for 72 hours and cell viability assays were performed. Fraction affected (Fa) values were calculated as described previously^45^. Fa values were used to evaluate drug combinations by CompuSyn software from ComboSyn, Inc. (Paramus, NJ, USA)^43^,^44^,^45^.

### Mice and tumor xenografts

For tumor xenografts, approximately, 1 × 10^6^ MOLT-4 cells in 100 μl RPMI-1640 were mixed with equal volume of Matrigel (BD Biosciences) and implanted subcutaneously into 6-week-old athymic nude mice bearing the nu/nu gene [NIH(s) (nu/nu)]. Mice were maintained in pathogen-free conditions. When the tumor sizes reached 150-200 mm^3^, mice were injected intra peritoneally with SQDG dissolved in PBS (2 mg/kg body weight/week), for two weeks. Tumor length (L) and width (W) were measured using Vernier calipers and tumor volume was calculated by the equation: Volume = L × W^2^/2 (mm^3^). Tumor doubling time (TD) was calculated and the overall response rate of xenografts to treatment was assessed by measuring specific tumor growth delay (SGD) as described previously^47^. Data were analyzed by student’s unpaired t-test and values were considered significantly different at *P* < 0.05 and expressed as the mean ± SEM.

### Immunohistochemistry

Immunohistochemistry of tumor tissues from SQDG and vehicle treated mice was performed as described by Jalava *et al.*^57^. Diaminobenzidine tetrahydrochloride (DAB) staining was done using DAB substrate kit (BD Pharmingen) and nuclei were stained with Harris’ hematoxyline solution for 30 seconds. Scoring of immunostaining was done as described by Perrone *et al.*^58^.

### Molecular docking

Crystal structure of the receptor molecule i.e. human topoisomerase I complexed with its substrate DNA and inhibitor camptothecin was collected from Protein Data Bank (PDB) (PDB ID: 1A36)^48^. Three-dimensional (3D) coordinates of DNA and camptothecin were removed from the 1A36 structure for docking of SQDG. Structure of the ligand sulfonoquinovosyl diacylglyceride (SQDG) was derived from Pubchem (CID 50898453)^20^ and 3D coordinates of the molecule were generated using Openbabel 2.3.1^59^. 3D structure of SQDG was optimized through energy minimization using AMBER force field of VEGAZZ 3.0.0.52 package^60^,^61^. Probable binding cavities of 1A36 were identified using the CASTp web server^62^ and molecular docking of SQDG with topo I was performed using the GOLD v5.0.1 package from Cambridge Crystallographic Data Centre^63^. Following parameters were used in the docking cycles: population size (100), selection pressure (1.100000), number of operations (100,000), number of islands (5), niche size (2), crossover weight (95), mutate weight (95), and migrate weight (10). 100 docking calculations were run for the ligand and the best docking solutions were identified based on lowest energy-docking mode and critical manual inspection satisfying favorable interactions between the ligand and the protein molecule.

## Additional Information

**How to cite this article**: Jain, C. K. *et al.* Sulfonoquinovosyl diacylglyceride selectively targets acute lymphoblastic leukemia cells and exerts potent anti-leukemic effects *in vivo*. *Sci. Rep.* **5**, 12082; doi: 10.1038/srep12082 (2015).

## Supplementary Material

Supplementary Information

## Figures and Tables

**Table 1:** Etoposide-SQDG combination effect analysis.

						Combination				
ETO (μM)	Cell viability %	Fa values for ETO	SQDG (μM)	Cell viability %	Fa values for SQDG	ETO (μM)	SQDG (μM)	Cell viability %	Fa values for combination	Combination index (CI) value
0.2	89.9 ± 2.7	0.111 ± 0.011	5	84.5 ± 3.2	0.166 ± 0.032	0.2	5	40.8 ± 2.7	0.483 ± 0.011	0.666 ± 0.017
0.4	79.8 ± 4.9	0.215 ± 0.031	10	68.5 ± 4.8	0.334 ± 0.022	0.4	10	27.3 ± 2.3	0.732 ± 0.020	0.760 ± 0.008
0.6	69.1 ± 7.6	0.349 ± 0.038	15	49.7 ± 1.0	0.507 ± 0.020	0.6	15	12.4 ± 5.2	0.868 ± 0.057	0.715 ± 0.135
0.8	58.1 ± 9.8	0.469 ± 0.008	20	38.6 ± 2.4	0.601 ± 0.036	0.8	20	6.8 ± 1.6	0.931 ± 0.016	0.667 ± 0.013
1.0	42.4 ± 5.2	0.584 ± 0.041	25	26.0 ± 4.2	0.732 ± 0.037	1.0	25	4.2 ± 0.6	0.957 ± 0.006	0.65 ± 0.058
1.2	32.8 ± 5.3	0.681 ± 0.043	30	14.7 ± 3.9	0.865 ± 0.038	1.2	30	1.9 ± 0.11	0.981 ± 0.001	0.516 ± 0.117
1.4	20.8 ± 1.9	0.779 ± 0.027	35	7.6 ± 4.5	0.937 ± 0.033	1.4	35	0.9 ± 0.2	0.99 ± 0.002	0.429 ± 0.113

MOLT-4 cells were treated with either SQDG or etoposide or etoposide-SQDG constant ratio combinations and cell viability data were analyzed using CompuSyn Software. Values are presented as mean ± SD, for the three independent experiments. ‘ETO’ is abbreviation for etoposide.

**Table 2:** Doxorubicin-SQDG combination effect analysis.

						Combination				
DOX (μM)	Cell viability %	Fa values for DOX	SQDG (μM)	Cell viability %	Fa values for SQDG	DOX (μM)	SQDG (μM)	Cell viability %	Fa values for combination	Combination index (CI) value
0.2	91.8 ± 1.0	0.081 ± 0.010	5	86.4 ± 0.5	0.135 ± 0.005	0.2	5	38.9 ± 2.4	0.559 ± 0.009	0.513 ± 0.032
0.4	82.7 ± 2.3	0.173 ± 0.022	10	70.3 ± 2.8	0.296 ± 0.028	0.4	10	23.1 ± 6.1	0.768 ± 0.061	0.624 ± 0.130
0.6	72.3 ± 7.3	0.276 ± 0.073	15	48.6 ± 1.7	0.513 ± 0.017	0.6	15	12.1 ± 1.9	0.878 ± 0.018	0.627 ± 0.063
0.8	62.3 ± 7.5	0.406 ± 0.124	20	38.8 ± 0.9	0.611 ± 0.008	0.8	20	5.0 ± 2.2	0.949 ± 0.021	0.501 ± 0.104
1.0	46.2 ± 3.1	0.534 ± 0.025	25	23.9 ± 0.5	0.760 ± 0.005	1.0	25	2.0 ± 0.6	0.979 ± 0.005	0.393 ± 0.034
1.2	37.0 ± 2.6	0.629 ± 0.025	30	16.0 ± 0.3	0.839 ± 0.003	1.2	30	1.4 ± 0.3	0.985 ± 0.003	0.404 ± 0.007
1.4	24.1 ± 4.1	0.758 ± 0.040	35	10.3 ± 2.9	0.897 ± 0.028	1.4	35	0.1 ± 0.1	0.998 ± 0.005	0.136 ± 0.036

MOLT-4 cells were treated with either SQDG or doxorubicin or doxorubicin-SQDG constant ratio combinations and cell viability data were analyzed using CompuSyn Software. Values are presented as mean ± SD, for the three independent experiments. ‘DOX’ is abbreviation for doxorubicin.
