# Supplementary material for: Sulfonoquinovosyl diacylglyceride selectively targets acute lymphoblastic leukemia cells and exerts potent anti-leukemic effects in vivo
Source: Sci Rep. 2015 Jul 20;5:12082. doi: 10.1038/srep12082 (PMC4507174; doi:10.1038/srep12082)
Supplement: Supplementary Information [file srep12082-s1.docx]

**Supplementary Information**

**Sulfonoquinovosyl diacylglyceride selectively targets acute lymphoblastic leukemia cells and exerts potent anti-leukemic effects *in vivo***

Chetan Kumar Jain^1, 2^, Bhola Shankar Pradhan^3^, Sukdeb Banerjee^4^, Nirup Bikash Mondal^4^, Subeer S. Majumder^3^, Madhumita Bhattacharyya^5^, Saikat Chakrabarti^5^, Susanta Roychoudhury^2*^, Hemanta Kumar Majumder^1*^.

**Supplementary Figures**


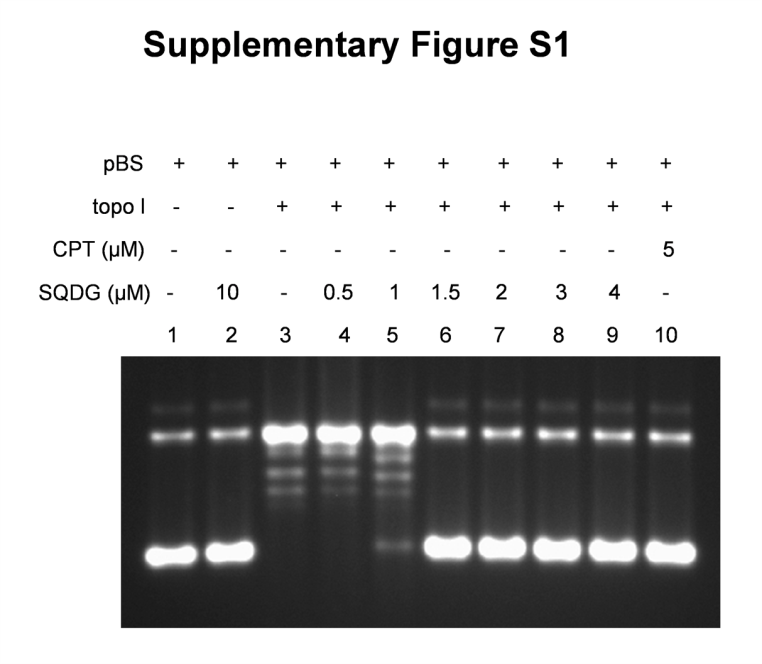


**Supplementary Figure S1.** Preincubation dilution DNA relaxation assay. Topo I was preincubated with indicated concentrations of SQDG or CPT for 5 minutes and then the reaction mixture was diluted to ten folds with the reaction buffer. After the dilution supercoiled pBS DNA was added. Reactions were incubated at 37 ^o^C for 30 minutes and relaxation assay was performed. Complete scan of the gel is presented in Supplementary Fig. S16.

**
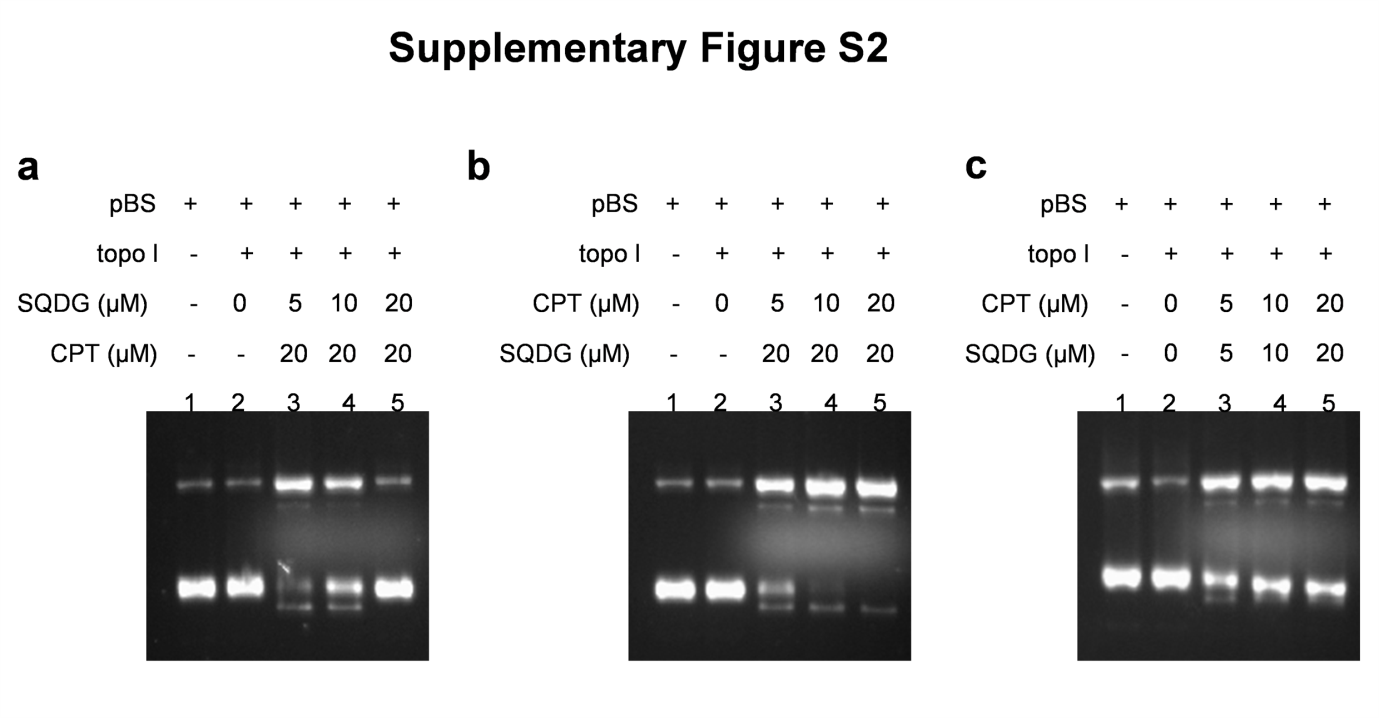
**

**Supplementary Figure S2.** Competition cleavage assay. (a) SQDG-topo I preincubation cleavage assay. Topo I was preincubated for 5 minutes with indicated concentrations of SQDG and then 20 µM CPT was added to the reactions. Supercoiled pBS DNA was added and cleavage assay was performed. (b) CPT-topo I preincubation cleavage assay. Topo I was preincubated for 5 minutes with indicated concentrations of CPT and then 20 µM SQDG was added to the reactions. Supercoiled pBS DNA was added and cleavage assay was performed. (c) SQDG/CPT simultaneous cleavage assay. SQDG and CPT both were added simultaneously to the reaction mixture at indicated concentrations. Supercoiled pBS DNA was added and cleavage assay was performed. Complete scans of the different gels are presented in Supplementary Fig. S17.

**
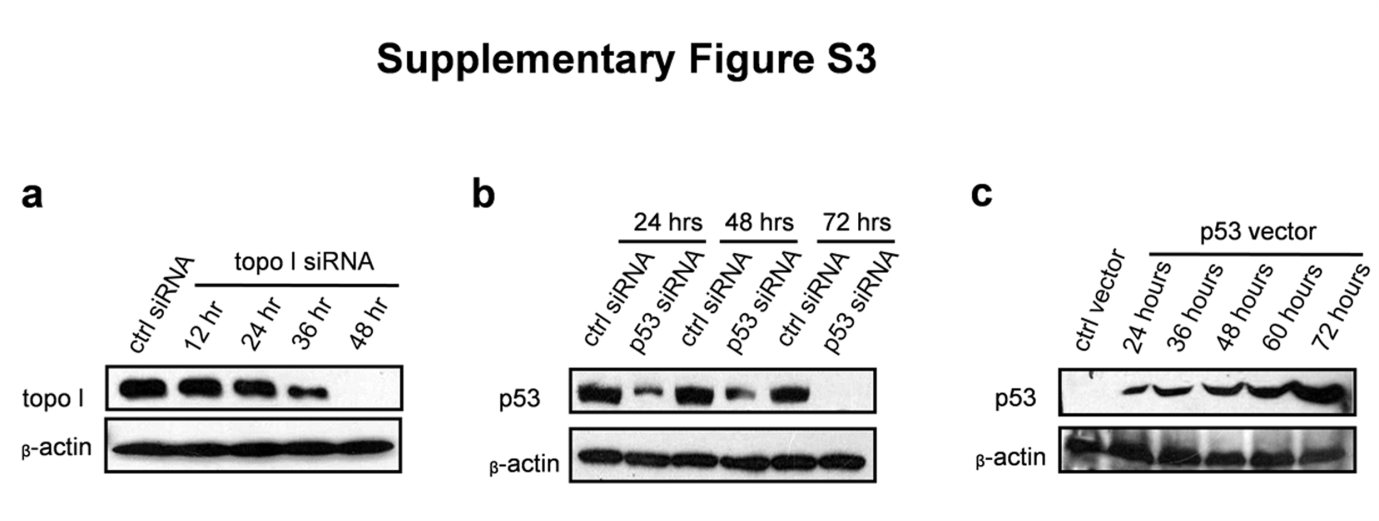
**

**Supplementary Figure S3.** Western blotting. (a) Western blot showing knockdown of topoisomerase I in MOLT-4 cells. MOLT-4 cells were transfected with 100 nM topo I siRNA or 100 nM control siRNA (ctrl siRNA) and harvested at 12, 24, 36 and 48 hours time points. (b) Western blot showing knockdown of p53 in MOLT-4 cells. MOLT-4 cells were transfected with 100 nM p53 siRNA or 100 nM control siRNA (ctrl siRNA) and harvested at 24, 48 and 72 hours time points. (c) Western blot showing ectopic expression of p53 in K562 cells. K562 cells were transfected with 400 ng control vector or 400 ng p53 expressing vector pCMV-NEO-BAM and harvested after 24, 36, 48, 60 and 72 hours time points. Complete scans of the different blots are presented in Supplementary Fig. S10 and S15.

**
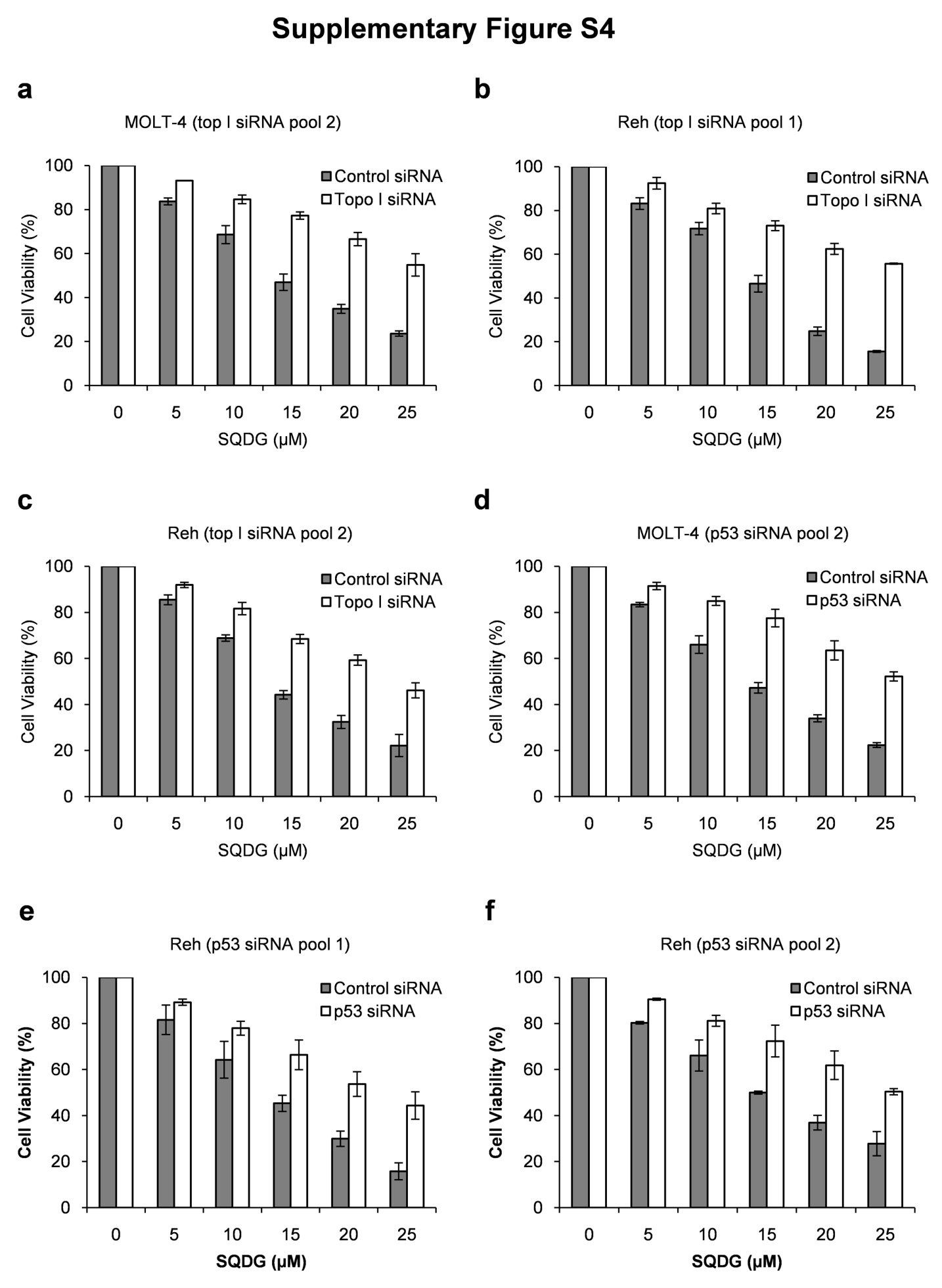
Supplementary Figure S4.** siRNA knockdown of topo I and p53 in MOLT-4 and Reh cell lines. (a) Knockdown of topo I in MOLT-4 cells by topo I siRNA pool 2. MOLT-4 cells were transfected with 100 nM topo I siRNA or 100 nM control siRNA. After 48 hours of transfection cells were treated with indicated concentrations of SQDG for 72 hours and MTT assay was performed. (b) and (c) Knockdown of topo I in Reh cells by topo I siRNA pool 1 and pool 2. Reh cells were transfected with 100 nM topo I siRNA or 100 nM control siRNA. After 48 hours of transfection cells were treated with indicated concentrations of SQDG for 72 hours and MTT assay was performed. (d) Knockdown of p53 in MOLT-4 cells by p53 siRNA pool 2. MOLT-4 cells were transfected with 100 nM p53 siRNA or 100 nM control siRNA. After 72 hours of transfection cells were treated with indicated concentrations of SQDG for 72 hours and MTT assay was performed. (e) and (f) Knockdown of p53 in Reh cells by p53 siRNA pool 1 and pool 2. Reh cells were transfected with 100 nM p53 siRNA or 100 nM control siRNA. After 72 hours of transfection cells were treated with indicated concentrations of SQDG for 72 hours and MTT assay was performed. Three independent experiments were performed and data are represented as mean % cell viability ± SD.

**
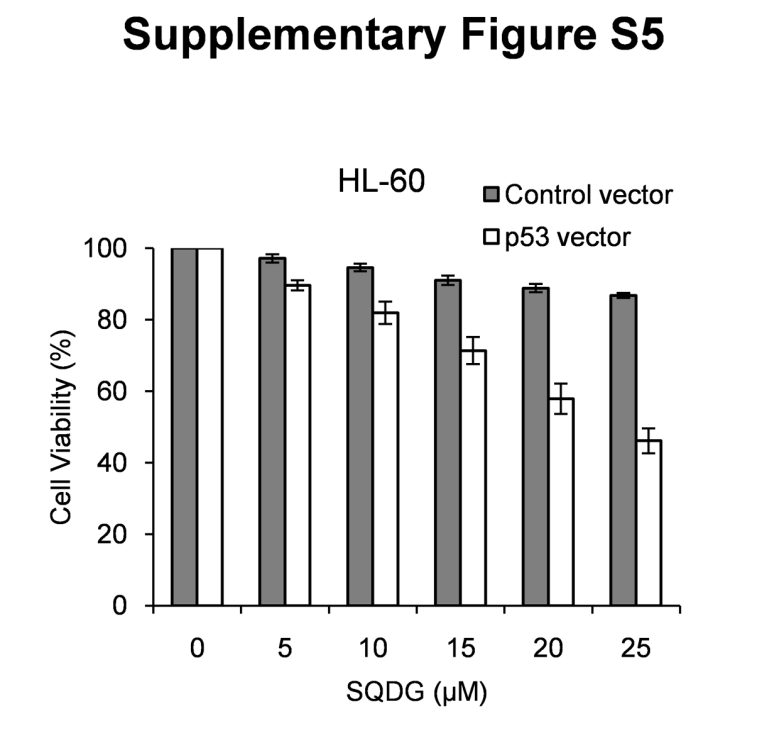
**

**Supplementary Figure S5.** Ectopic expression of p53 in p53 deficient HL-60 cells. HL-60 cells were transfected with 400 ng control vector or 400 ng p53 expressing vector pCMV-NEO-BAM. After 36 hours of transfection cells were treated with indicated concentrations of SQDG for 72 hours and MTT assay was performed. Three independent experiments were performed and data are represented as mean % cell viability ± SD. Hollow bars indicate cells transfected with control vector and solid bars indicate cells transfected with p53 expressing vector.

**
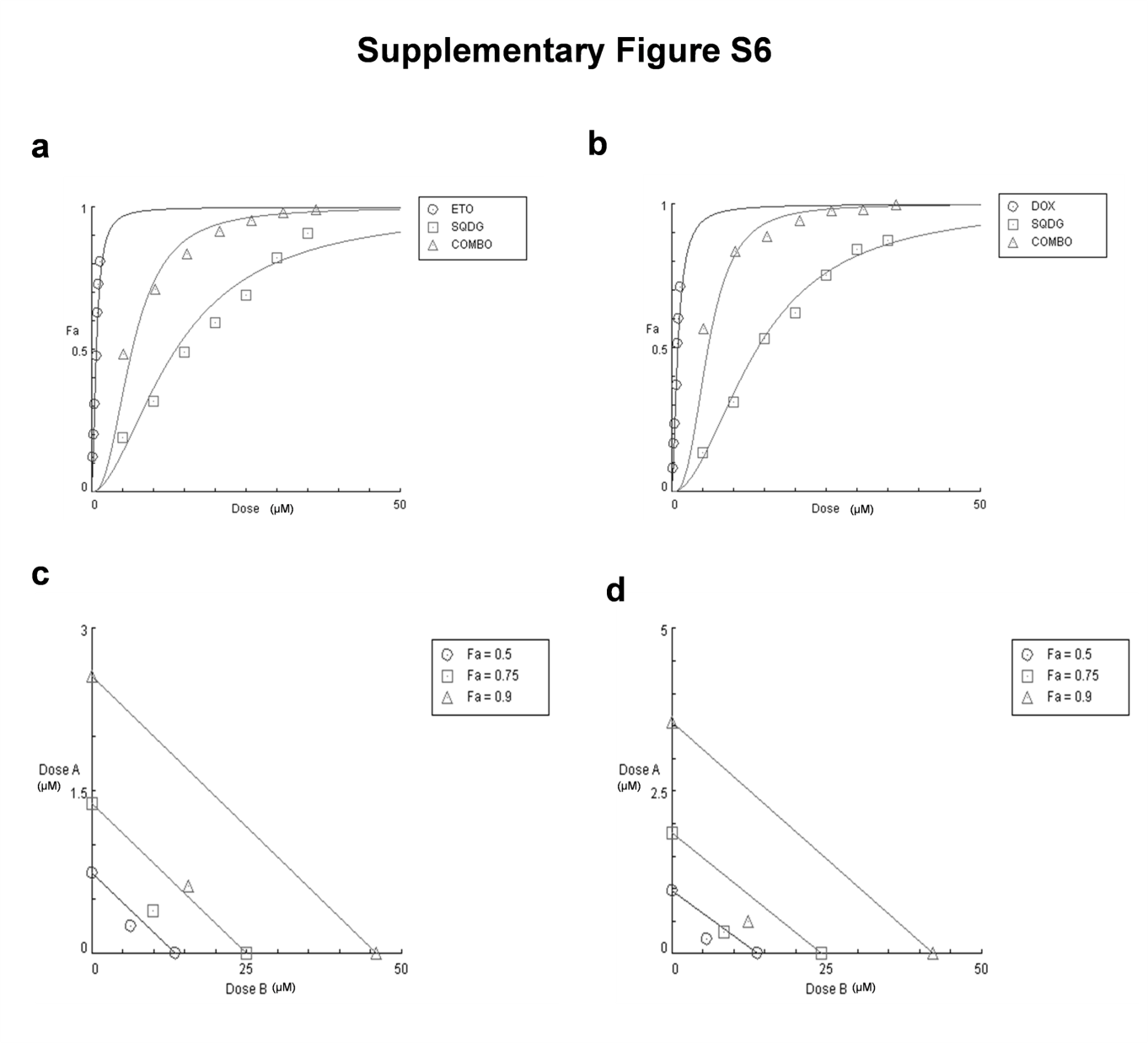
**

**Supplementary Figure S6.** Combination effect analysis of etoposide-SQDG and doxorubicin-SQDG constant ratio combinations using CompuSyn software. (a) Dose effect curve for etoposide (ETO), SQDG and ETO-SQDG combination. On X-axis ‘Dose’ represents doses of ETO or SQDG or ETO-SQDG combination. On Y-axis ‘Fa’ represents fraction affected values. ‘COMBO’ denotes for the combination. (b) Dose effect curve for doxorubicin (DOX), SQDG and DOX-SQDG combination. On X-axis ‘Dose’ represents doses of DOX or SQDG or DOX-SQDG combination. On Y-axis ‘Fa’ represents fraction affected values. ‘COMBO’ denotes for the combination. (c) Isobologram for ETO, SQDG and ETO-SQDG combination. On X-axis dose B represents SQDG dose concentrations in µM. On Y-axis dose A represents ETO dose concentrations in µM. (d) Isobologram for DOX, SQDG and DOX-SQDG combination. On X-axis dose B represents SQDG dose concentrations in µM. On Y-axis dose A represents DOX dose concentrations in µM.

**
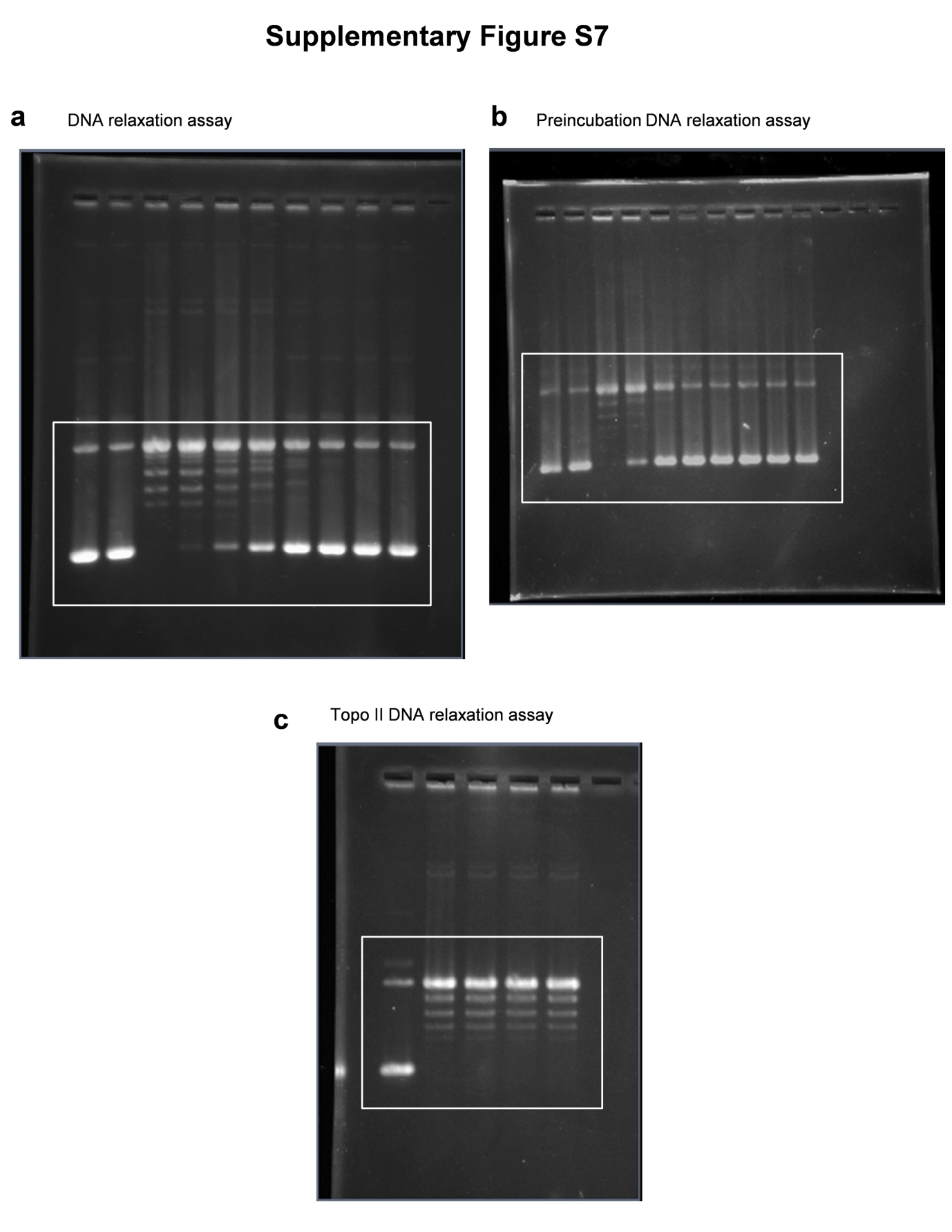
 Supplementary Figure S7.** SQDG inhibits relaxation activity of human topoisomerase I enzyme. (a) DNA relaxation assay of topo I enzyme. (b) Preincubation DNA relaxation assay. (c) DNA relaxation assay of topo II enzyme.

**
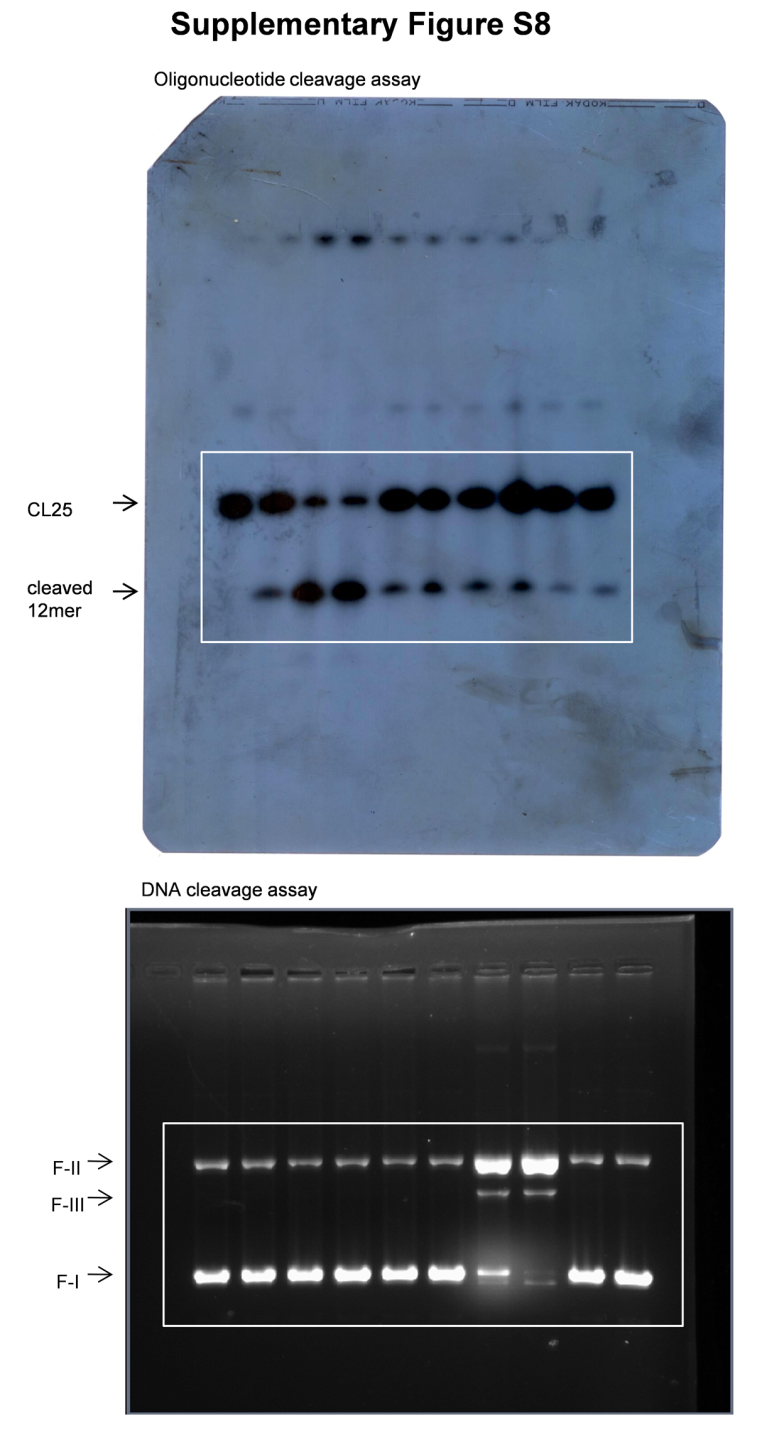
**

**Supplementary Figure S8.** SQDG abrogates CPT mediated topo I-DNA complex formation *in vitro*. Oligonucleotide cleavage assay (upper image). Plasmid DNA cleavage assay (lower image).

**
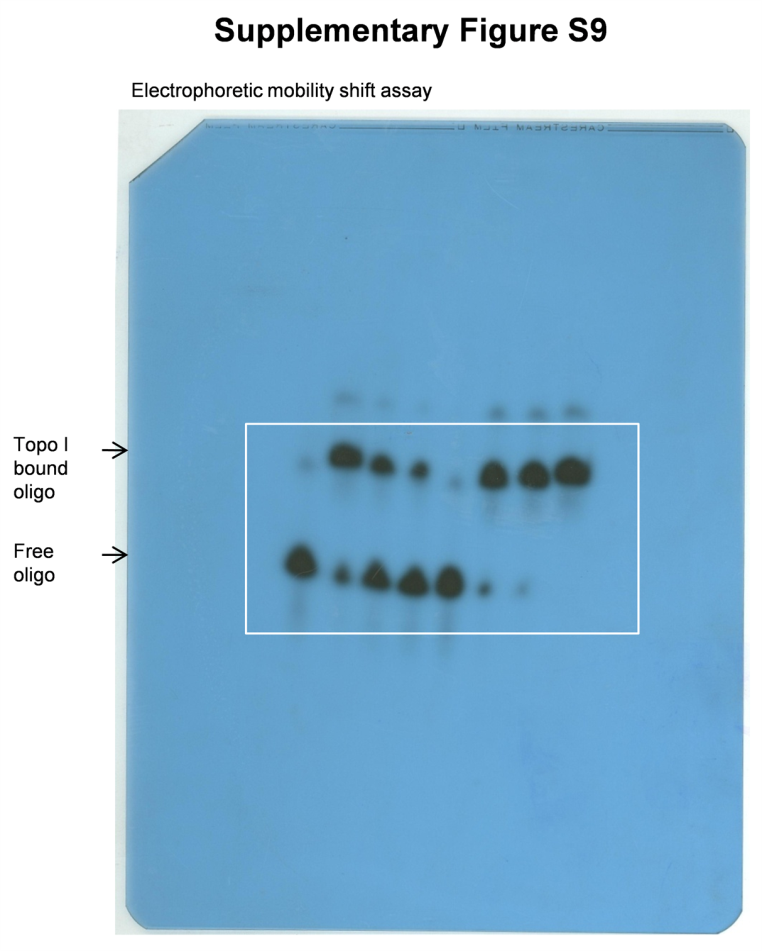
**

**Supplementary Figure S9.** Electrophoretic mobility shift assay. Lane 1, 1 pmol of the labeled 25-mer duplex oligo; lane 2, 1 pmol of the labeled 25-mer duplex oligo with 20 ng topo I enzyme; lanes 3 to 8 are same as lane 2 but in the presence of indicated concentrations of SQDG and CPT.

**
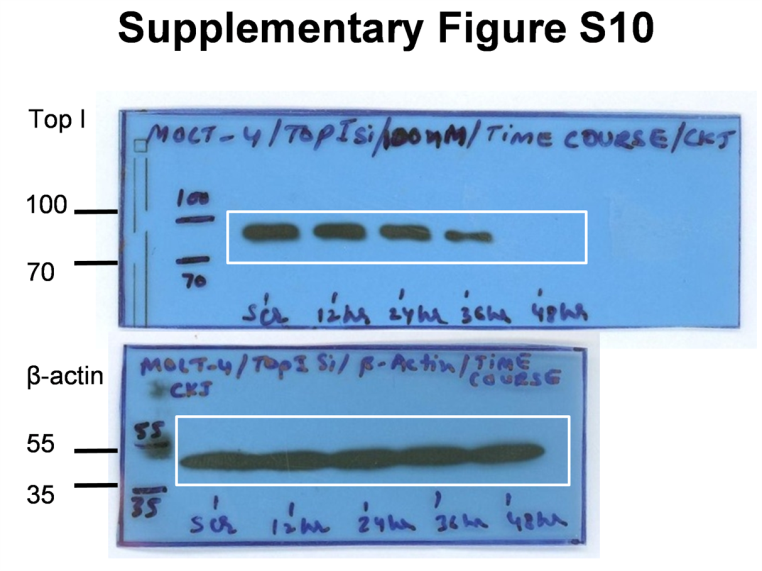
**

**Supplementary Figure S10.** Western blot showing knockdown of topoisomerase I in MOLT-4 cells. MOLT-4 cells were transfected with 100 nM topo I siRNA or 100 nM control siRNA (ctrl siRNA) and harvested at 12, 24, 36 and 48 hours time points.

**
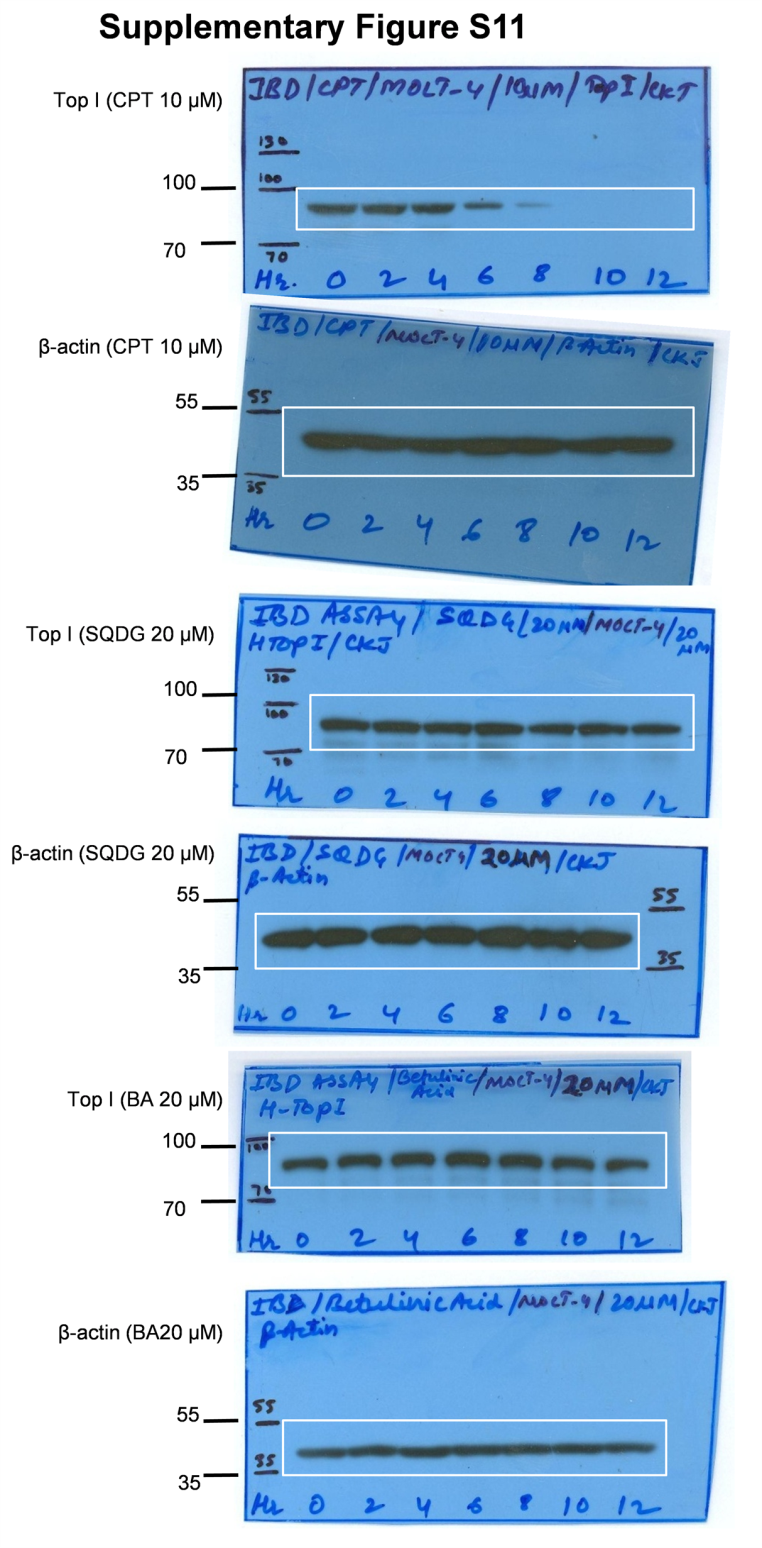
**

**Supplementary Figure S11.** Topo I immunoband depletion assay in MOLT-4 cells. The cells were treated with 10 µM CPT or 20 µM SQDG or 20 µM BA and harvested at indicated time points. Western blotting was performed using anti-topo I or anti-β-actin antibodies.

**
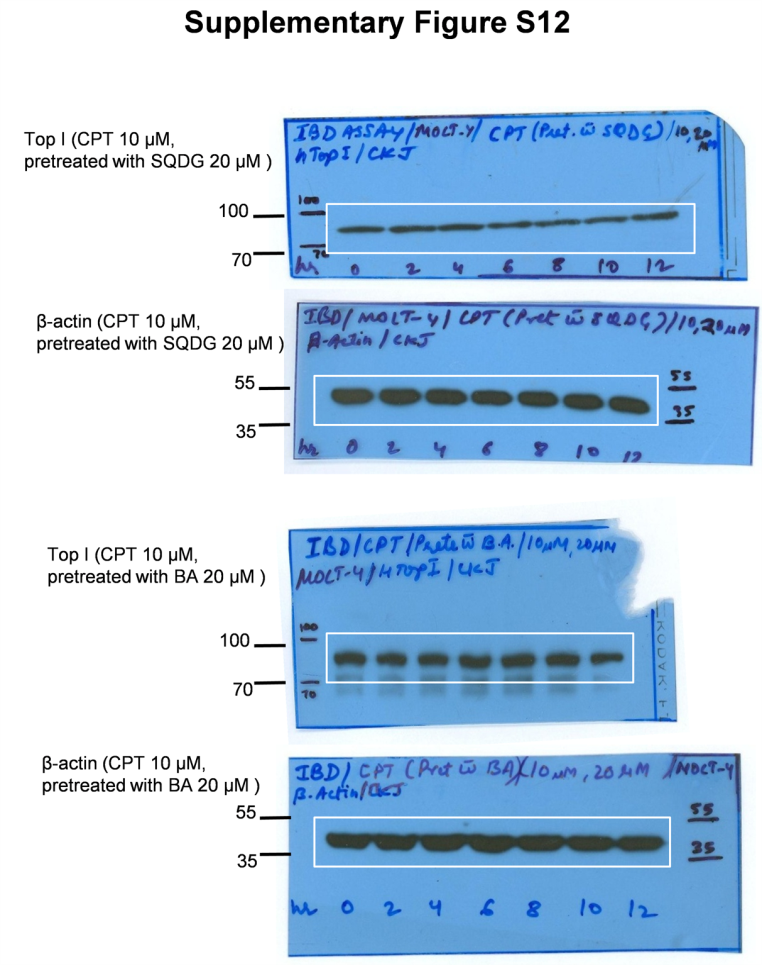
**

**Supplementary Figure S12.** Pretreatment immunoband depletion assay. MOLT-4 cells were first treated with either 20 µM SQDG or 20 µM BA for 2 hours and then treated with 10 µM CPT for indicated time points. Western blotting was performed using anti-topo I or anti-β-actin antibodies.

**
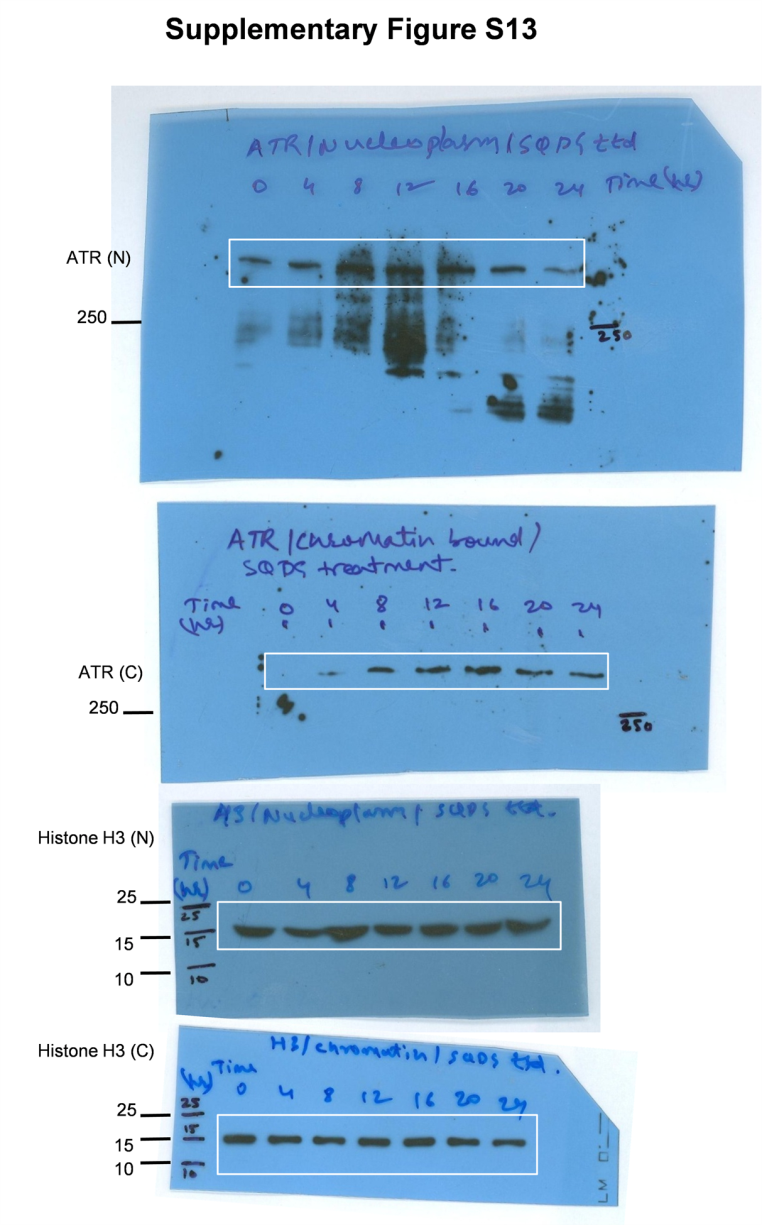
**

**Supplementary Figure S13.** ATR recruitment at chromatin in SQDG treated MOLT-4 cells. MOLT-4 cells were treated with 15 µM SQDG for indicated time points and nuclear and chromatin fractionations were performed. Levels of ATR in chromatin and nuclear fractions were detected by western blot analysis. Histone-H3 was used as loading control. ‘N’ stands for nuclear fraction and ‘C’ stands for chromatin fraction.

**
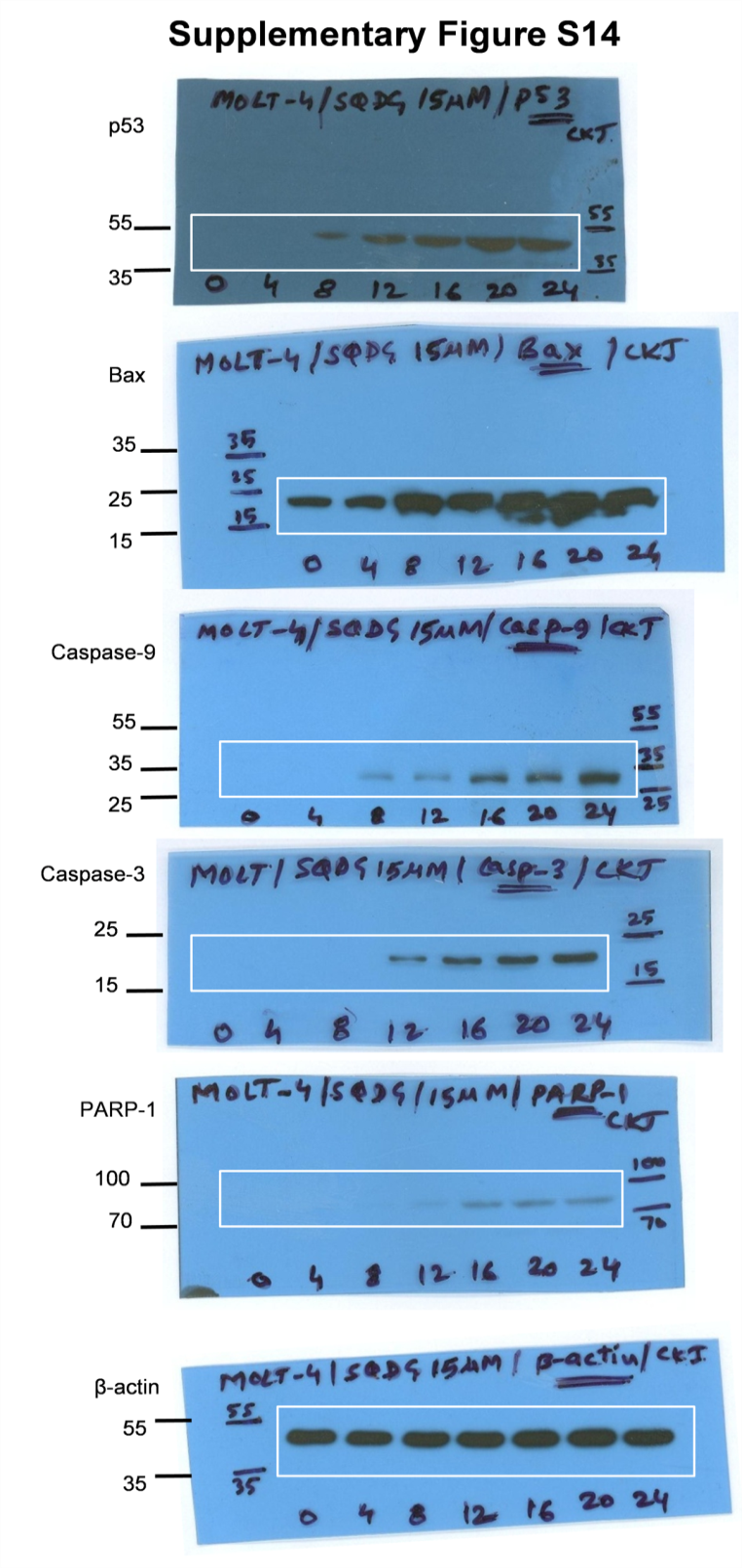
**

**Supplementary Figure S14.** Analysis of p53 dependent apoptotic pathway. MOLT-4 cells were treated with 15 µM SQDG for indicated time points and immunoblotting was performed using specific antibodies for indicated proteins involved in p53 dependent pathway.

**
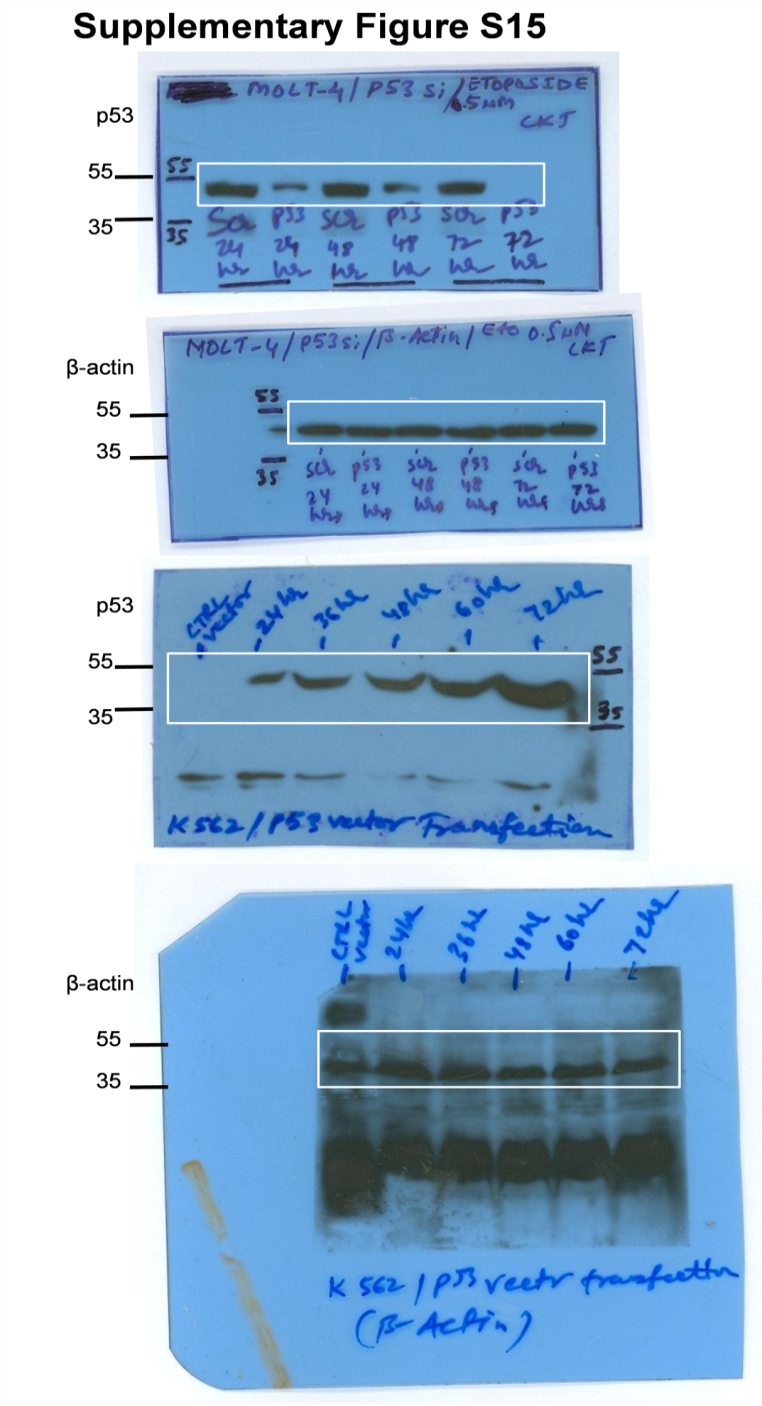
**

**Supplementary Figure S15.** Western blots (upper two) showing knockdown of p53 in MOLT-4 cells. MOLT-4 cells were transfected with 100 nM p53 siRNA or 100 nM control siRNA (ctrl siRNA) and harvested at 24, 48 and 72 hours time points. Lower two western blots showing ectopic expression of p53 in K562 cells. K562 cells were transfected with 400 ng control vector or 400 ng p53 expressing vector pCMV-NEO-BAM and harvested after 24, 36, 48, 60 and 72 hours time points.

**
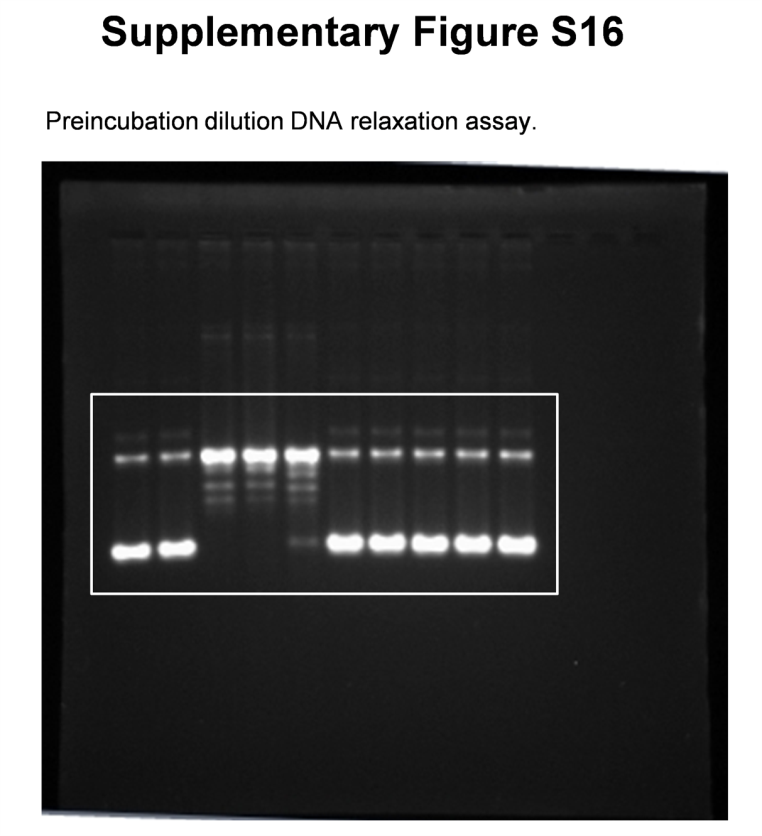
**

**Supplementary Figure S16.** Preincubation dilution DNA relaxation assay. Topo I was preincubated with indicated concentrations of SQDG or CPT for 5 minutes and then the reaction mixture was diluted to ten folds with the reaction buffer. After the dilution supercoiled pBS DNA was added. Reactions were incubated at 37 ^o^C for 30 minutes and relaxation assay was performed.

**
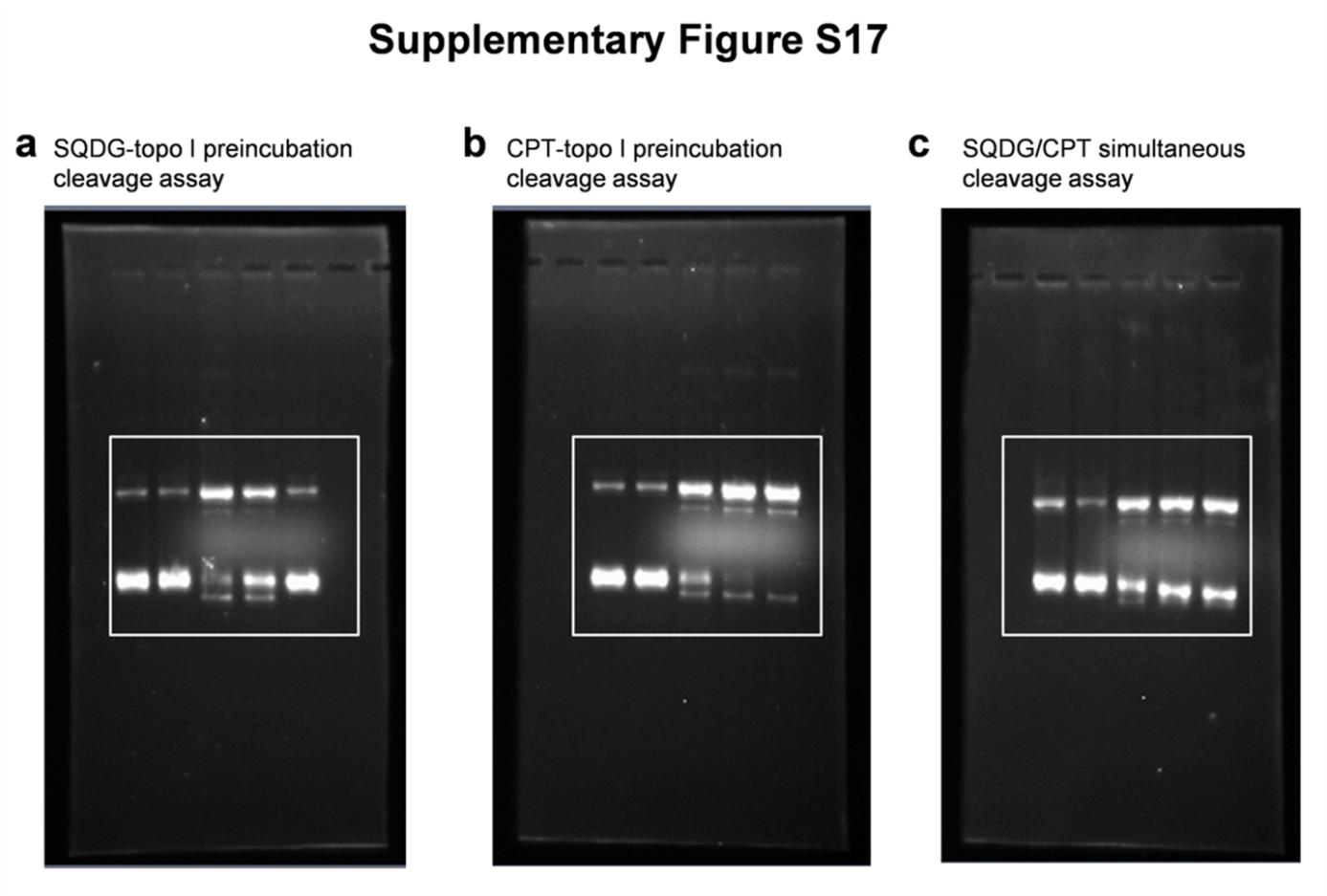
 Supplementary Figure S17.** Competition cleavage assay. (a) SQDG-topo I preincubation cleavage assay. Topo I was preincubated for 5 minutes with indicated concentrations of SQDG and then 20 µM CPT was added to the reactions. Supercoiled pBS DNA was added and cleavage assay was performed. (b) CPT-topo I preincubation cleavage assay. Topo I was preincubated for 5 minutes with indicated concentrations of CPT and then 20 µM SQDG was added to the reactions. Supercoiled pBS DNA was added and cleavage assay was performed. (c) SQDG/CPT simultaneous cleavage assay. SQDG and CPT both were added simultaneously to the reaction mixture at indicated concentrations. Supercoiled pBS DNA was added and cleavage assay was performed.

**Supplementary Tables**

**Supplementary Table S1.** Effect of SQDG treatment on different cell lines. Cells were treated with different concentrations of SQDG for 72 hours and cell viability assays were performed (wt = wild type, mt = mutant).

| Cell line | p53 status | IC_50_ or effect of SQDG on cell killing |
| --- | --- | --- |
| MOLT-4 | wt | IC_50_ = 15.32 ± 0.58 μM |
| MOLT-3 | wt | IC_50_ = 22.52 ± 0.64 μM |
| Reh | wt | IC_50_ = 19.63 ± 0.23 μM |
| Jurkat | mt | IC_50_ = 75.67 ± 6.4 μM |
| U937 | mt | Partially affected (~20% cell killing at 50 μM) |
| THP-1 | mt | No cell killing |
| RAJI | mt | No cell killing |
| K562 | No protein expression | Partially affected (~18% cell killing at 50 μM) |
| HL-60 | No protein expression | No cell killing |
| HCT116 | wt | No cell killing |
| A549 | wt | Partially affected (~25% cell killing at 50 μM) |
| HepG2 | wt | No cell killing |
| SW480 | mt | No cell killing |
| SKBR-3 | mt | No cell killing |
| MIAPaCa | mt | No cell killing |
| WI-38 | wt | No cell killing |
| PBMC | wt | No cell killing |

**Supplementary Table S2.** Differential sensitivity of MOLT-4 and Reh cell lines for SQDG upon siRNA silencing of *TOP1* gene.

| Topo I siRNA | MOLT-4 | | | Reh | | |
| --- | --- | --- | --- | --- | --- | --- |
|  | Control siRNA (µM) | Topo I silenced (µM) | Fold sensitivity | Control siRNA (µM) | Topo I silenced (µM) | Fold sensitivity |
| siRNA pool 1 | 14.04 ± 0.71 | 29.09 ± 2.08 | 2.07 | 14.48 ± 0.28 | 27.51 ± 0.41 | 1.89 |
| siRNA pool 2 | 15.56 ± 0.33 | 29.09 ± 1.67 | 1.86 | 15.22 ± 0.61 | 23.84 ± 1.31 | 1.57 |

**Supplementary Table S3.** Effect of SQDG treatment on the cell cycle phase distribution of MOLT-4 cells.

| Treatment | Cell cycle stages | | |
| --- | --- | --- | --- |
|  | G_1_ (%) | S (%) | G_2_/M (%) |
| Mock | 77.2 | 8.7 | 14.0 |
| 15 µM SQDG | 53.0 | 42.2 | 4.8 |
| 20 µM SQDG | 49.4 | 45.2 | 5.4 |
| 25 µM SQDG | 43.6 | 50.4 | 6.1 |

**Supplementary Table S4.** Recruitment of ATR at chromatin upon SQDG treatment.

| SQDG treatment time (hours) | ATR in nuclear fraction  (average fold change) | ATR in chromatin fraction  (average fold change) |
| --- | --- | --- |
| 0 | 1 | 1 |
| 4 | 1.88 ± 0.07 | 23.15 ± 0.16 |
| 8 | 6.83 ± 2.26 | 200.78 ± 17.49 |
| 12 | 3.73 ± 0.15 | 240.13 ± 25.2 |
| 16 | 3.1 ± 0.55 | 340.3 ± 34.42 |
| 20 | 1.34 ± 0.04 | 216.07 ± 30.56 |
| 24 | 0.49 ± 0.12 | 196.57 ± 30.84 |

**Supplementary Table S5.** Differential Sensitivity of MOLT-4 and Reh cell lines for SQDG upon siRNA silencing of *TP53* gene.

| p53 siRNA | MOLT-4 | | | Reh | | |
| --- | --- | --- | --- | --- | --- | --- |
|  | Control siRNA (µM) | p53 silenced (µM) | Fold sensitivity | Control siRNA (µM) | p53 silenced (µM) | Fold sensitivity |
| siRNA pool 1 | 15.15 ± 0.13 | 26.85 ± 2.08 | 1.77 | 14.31 ± 1.04 | 22.35 ± 2.42 | 1.56 |
| siRNA pool 2 | 15.28 ± 0.4 | 27.49 ± 1.47 | 1.79 | 16.05 ± 1.06 | 25.9 ± 2.31 | 1.61 |

**Supplementary Table S6.** Diffferential sensitivity of K562 and HL-60 cell lines for SQDG upon ectopic expression of p53.

| K562 | | | HL-60 | | |
| --- | --- | --- | --- | --- | --- |
| Control vector (µM) | p53 vector (µM) | Fold sensitivity | Control vector (µM) | p53 vector (µM) | Fold sensitivity |
| 56.51 ± 1.14 | 19.54 ± 2.51 | 2.89 | 92.65 ± 6.35 | 24.01 ± 2.0 | 3.85 |

**Supplementary Table S7.** Details of the antibodies used.

| Antibody name | Manufacturer | Catalogue number |
| --- | --- | --- |
| Topo I | Santa Cruz Biotechnology | Sc-271285 |
| β-actin | Sigma | A2228 SIGMA |
| ATR | Cell Signalling Technology | 2790 |
| Histone H3 | Santa Cruz Biotechnology | Sc-10809 |
| p53 | Santa Cruz Biotechnology | Sc-126 |
| Bax | Santa Cruz Biotechnology | Sc-70406 |
| Cleaved caspase-9 | Cell Signalling Technology | 7237 |
| Cleaved caspase-3 | Cell Signalling Technology | 9664 |
| PARP-1 | Cell Signalling Technology | 5625 |
| Phospho-Histone H2AX (Ser-139) | Cell Signalling Technology | 9718 |
| Cyclin A2 | Santa Cruz Biotechnology | Sc-751 |
| Ki-67 | Santa Cruz Biotechnology | Sc-23900 |

**Supplementary Table S8.** Details of the siRNAs used.

| siRNA name | Manufacturer | Catalogue number |
| --- | --- | --- |
| Topo I (pool 1) | Santa Cruz Biotechnology | Sc-36694 |
| Topo I (pool 2) | Ambion, Invitrogen | AM16708 |
| p53 (pool 1) | Ambion, Invitrogen | AM16210, Custom made |
| p53 (pool 2) | Dharmacon | M-003329-03-0005 |
| Scrambled siRNA | Ambion | Ambion |

**Supplementary Table S9.** Immunohistochemical analysis of different pharmacodynamic markers in vehicle and SDQG treated tumor tissues.

| Marker | Treatment | Quantitative evaluation | | Staining intensity | | Final evaluation | |
| --- | --- | --- | --- | --- | --- | --- | --- |
|  |  | % immunopositive cells | Score | Intensity | Score | Combined score | Expression |
| PARP-1 | Vehicle | 0.5 ± 0.09 | 0 | Weak | 1 | 1 | Low |
|  | SQDG | 21.26 ± 6.37 | 2 | Strong | 3 | 5 | Intermediate |
| Cyclin A2 | Vehicle | 9.19 ± 2.47 | 1 | Moderate | 2 | 3 | Intermediate |
|  | SQDG | 55.53 ± 2.09 | 3 | Strong | 3 | 6 | High |
| γ-H2AX | Vehicle | 0.81 ± 0.41 | 0 | Moderate | 2 | 2 | Low |
|  | SQDG | 21.45 ± 2.17 | 2 | Strong | 3 | 5 | Intermediate |
| Ki-67 | Vehicle | 66.52 ± 14.11 | 3 | Strong | 3 | 6 | High |
|  | SQDG | 1.89 ± 0.38 | 1 | Weak | 1 | 2 | Low |
